# Decreased MFN2 activates the cGAS-STING pathway in diabetic myocardial ischaemia–reperfusion by triggering the release of mitochondrial DNA

**DOI:** 10.1186/s12964-023-01216-y

**Published:** 2023-08-03

**Authors:** Yonghong Xiong, Yan Leng, Hao Tian, Xinqi Deng, Wenyuan Li, Wei Li, Zhongyuan Xia

**Affiliations:** Department of Anesthesiology, Renmin Hospital of Wuhan University, Wuhan, China

**Keywords:** Diabetes, Myocardial ischaemia–reperfusion, Mitochondrial DNA, cGAS-STING

## Abstract

**Background:**

The cause of aggravation of diabetic myocardial damage is yet to be elucidated; damage to mitochondrial function has been a longstanding focus of research. During diabetic myocardial ischaemia–reperfusion (MI/R), it remains unclear whether reduced mitochondrial fusion exacerbates myocardial injury by generating free damaged mitochondrial DNA (mitoDNA) and activating the cGAS-STING pathway.

**Methods:**

In this study, a mouse model of diabetes was established (by feeding mice a high-fat diet (HFD) plus a low dose of streptozotocin (STZ)), a MI/R model was established by cardiac ischaemia for 2 h and reperfusion for 30 min, and a cellular model of glycolipid toxicity induced by high glucose (HG) and palmitic acid (PA) was established in H9C2 cells.

**Results:**

We observed that altered mitochondrial dynamics during diabetic MI/R led to increased mitoDNA in the cytosol, activation of the cGAS-STING pathway, and phosphorylation of the downstream targets TBK1 and IRF3. In the cellular model we found that cytosolic mitoDNA was the result of reduced mitochondrial fusion induced by HG and PA, which also resulted in cGAS-STING signalling and activation of downstream targets. Moreover, inhibition of STING by H-151 significantly ameliorated myocardial injury induced by MFN2 knockdown in both the cell and mouse models. The use of a fat-soluble antioxidant CoQ10 improved cardiac function in the mouse models.

**Conclusions:**

Our study elucidated the critical role of cGAS-STING activation, triggered by increased cytosolic mitoDNA due to decreased mitochondrial fusion, in the pathogenesis of diabetic MI/R injury. This provides preclinical insights for the treatment of diabetic MI/R injury.

Video Abstract

**Graphical Abstract:**

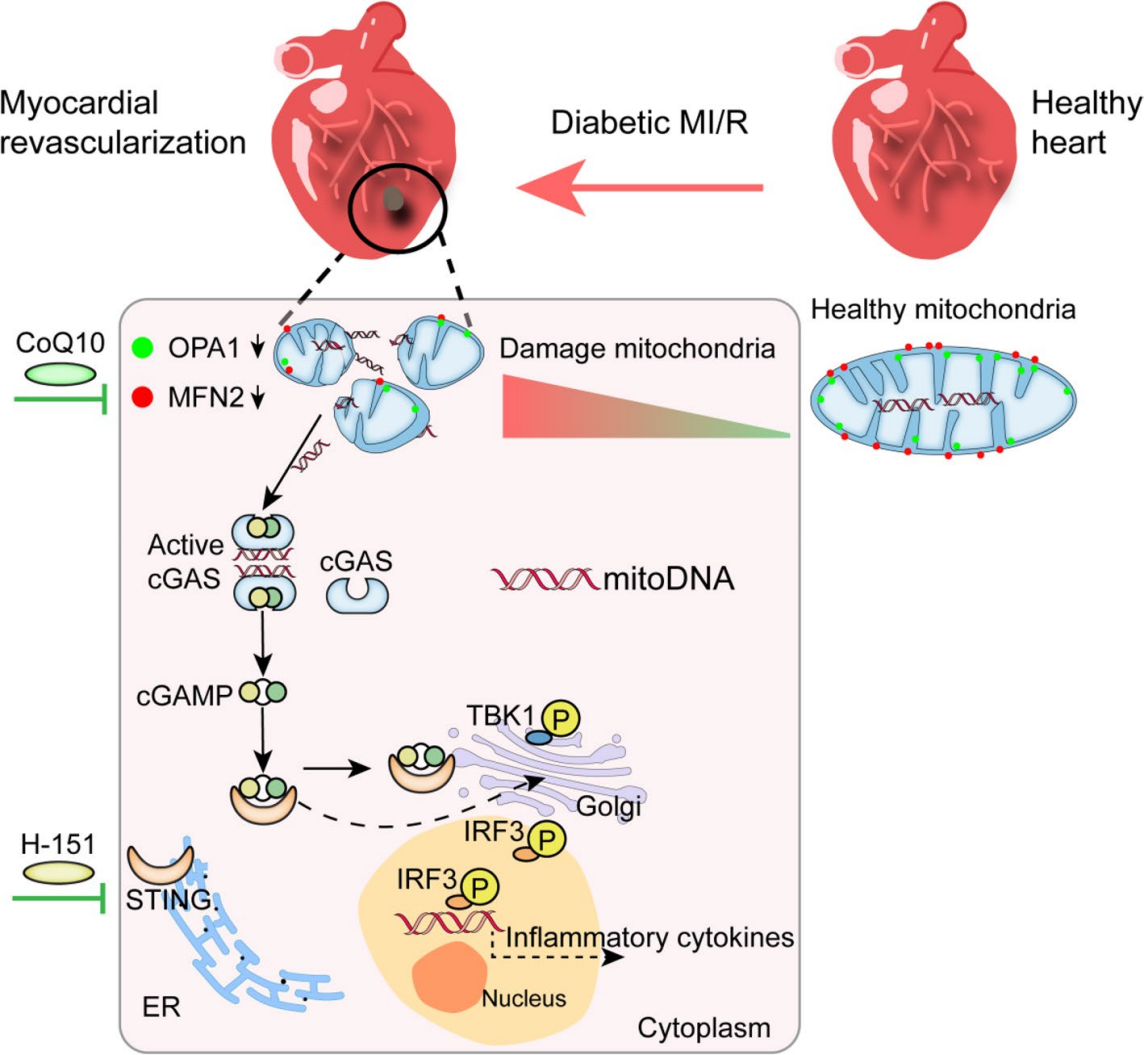

**Supplementary Information:**

The online version contains supplementary material available at 10.1186/s12964-023-01216-y.

## Background

Type 2 diabetes (T2D) is a global threat that affects millions of people worldwide [1]. T2D is an important risk factor for myocardial infarction, leading to severe left ventricular dysfunction after myocardial revascularisation, increasing the risk of ischaemia–reperfusion (I/R) injury, and complicating disease progression [2, 3]. T2D alters lipid metabolism, increases the production of reactive oxygen species, and increases systemic inflammation through the release of free fatty acids and inflammatory mediators. There is no evidence that the indications for myocardial revascularisation differ between patients with and without diabetes [4]. Current guidelines recommend the use of the same principles of myocardial revascularisation in diabetic and non-diabetic patients [5]. Moreover, ischaemic preconditioning and many cardioprotective pharmacological agents fail to exert their protective effects in diabetic ischaemic hearts [6]. Therefore, reducing the susceptibility of diabetic myocardial infarction to reperfusion injury is of great clinical value.

Indeed, previous studies have reported the mechanisms underlying the increased susceptibility to myocardial ischaemia–reperfusion (MI/R) injury in T2D, including that T2D itself triggers hyperglycaemia and insulin resistance [7], as well as the release of oxidative stress and inflammation induced during ischaemia and reperfusion [8], both of which lead to mitochondrial dysfunction [7, 8]. Despite increasing evidence of a relationship between mitochondrial dysfunction and its role in the pathophysiology of MI/R in T2D, effective therapeutic strategies to combat the disease by targeting the mitochondria are yet to be implemented. Therefore, identifying the molecular basis linking diabetes and MI/R injury is scientifically important and may provide effective therapeutic approaches. Recent studies have highlighted the crucial role of changes in mitochondrial morphology in mitochondrial dysfunctions. Mitochondrial morphological changes, also referred to as mitochondrial dynamics, primarily involve mitochondrial fusion and fission. Continuous cycles of fusion and fission determine mitochondrial morphology and regulate various mitochondrial functions, including energy production, oxidative stress, and inflammatory responses [9]. Mitochondrial outer membrane fusion is regulated by mitofusins 1 and 2 (MFN1 and 2); inner membrane fusion is regulated by optic atrophy 1 (OPA1), and mitochondrial fission is regulated by fission protein 1 (FIS1) and dynamin-related protein-1 (DRP1) [9]. Together, these proteins regulate the dynamic mitochondrial homeostasis.

Cyclic GMP-AMP (cGAMP) synthase (cGAS), in conjunction with double-stranded DNA, stimulates the conversion of ATP and GTP to cGAMP, which in turn triggers the stimulation of the interferon gene (STING) localised in the endoplasmic reticulum [10]. Ischaemic disease can damage and release endogenous DNA, including nuclear DNA (nDNA) and mitochondrial DNA (mitoDNA), into the cytoplasm [11], activate the cGAS-STING pathway, translocate STING from the endoplasmic reticulum to the Golgi, activate TBK1 and IRF3 phosphorylation, and trigger a sterile inflammatory response that exacerbates tissue damage [12, 13]. Circulating mitoDNA has been identified as a potential biomarker of I/R damage in the gut [14]. Recent studies have shown that circulating free nDNA and mitoDNA are involved in the pathophysiological process of many diseases as important damage-associated molecular patterns (DAMPs), including diabetic myocardial hypertrophy [15], acute kidney injury [16], sickle cell disease [17], and others.

In conclusion, it is worth investigating whether alterations in mitochondrial fusion are key to cytoplasmic enrichment of mitoDNA in diabetic MI/R injury. We hypothesised that mitochondrial dynamics are altered in diabetic MI/R injury, with reduced fusion and increased division leading to mitoDNA leakage and accumulation in the cytoplasm, activation of the cGAS-STING pathway, and a series of inflammatory responses that exacerbate tissue damage.

## Methods

### Reagents and antibodies

Streptozocin (STZ) and Palmitate (PA) were purchased from Sigma-Aldrich (St. Louis, MO, USA). The lactate dehydrogenase (LDH) cytotoxicity assay kit and BCA protein assay kit were obtained from Cell Signalling Technology (Danvers, MA, USA), while the malondialdehyde (MDA) content assay kit was purchased from MyBioSource (San Diego, CA, USA). Assay kits for COX I, COX II, and COX V activity were obtained from Abcam (Cambridge, UK). The XF Cell Mito Stress Test Kit was purchased from Agilent Technologies (Palo Alto, CA, USA). Assay kits for ATP content, tissue mitochondria isolation, and cell mitochondria isolation were purchased from Absin (Shanghai, China). rAAV-MFN2 and rAAV-OPA1 were obtained from Sangon Biotech (Shanghai, China). pLV3-U6-MFN2(rat)-shRNA-EGFP and pLV3-U6-OPA1(rat)-shRNA-EGFP were purchased from MiaolingBio (Wuhan, Hubei, China). Coenzyme Q10 (CoQ10) was obtained from MACKLIN (Shanghai, China). H-151 was obtained from MedChemExpress (Shanghai, China). 2,3,5-Triphenyte-trazoliumchloride (TTC) and Evans Blue dye were purchased from ServiceBio (Wuhan, Hubei, China). Antibodies against cGAS, PTEN-induced kinase 1 (PINK1), and type I interferon regulatory factor 3 (IRF3) were obtained from Thermo Fisher Scientific (Waltham, MA, USA). Antibodies against MFN2, OPA1, DRP1, STING, TANK-binding kinase 1 (TBK1), phospho-TBK1 (p-TBK1), phospho-IRF3 (p-IRF3), parkin, microtubule-associated protein light chain 3 II (LC3 II), human genes encoding p62/SQSTM1 (p62), lysosome-associated membrane protein 2 (Lamp2), cytochrome c (Cyto c), and anti-glyceraldehyde 3-phosphate dehydrogenase (GAPDH) were obtained from Cell Signaling Technology (Danvers, MA, USA). Anti-8-OHdG was purchased from Santa Cruz Biotechnology (Dallas, TX, USA). Unspecified reagents were purchased from ServiceBio (Wuhan, Hubei, China).

### Experimental animals and treatments

**Fig. 1:**
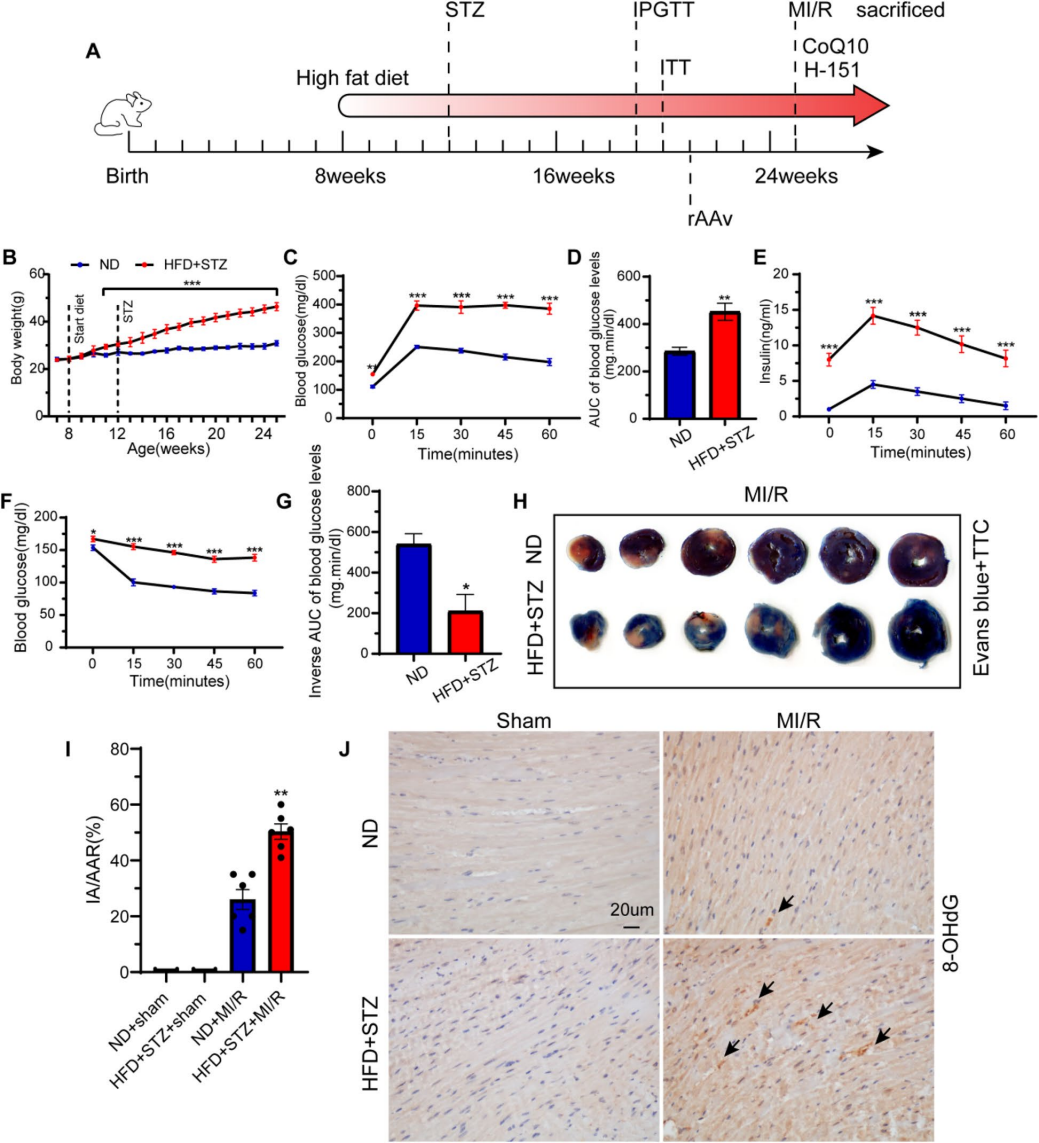
Diabetic model successfully, MI/R injury increased damaged DNA. **A** Animal experiment process. **B** Body weight of ND-fed and HFD-fed. C57BL/6 J mice were set on either 60% HFD, or normal diet to serve as a control. Results are mean ± SEM. ^***^ P < 0.001, *n* = 50. **C** Glucose levels during IPGTT. Results are mean ± SEM. ^**^ P < 0.01, ^***^ P < 0.001. *n* = 50. **D** Glucose area under the curve (AUC) during IPGTT. The blood glucose level measured at each time period subtracted the baseline blood glucose gave the corrected AUC. An AUC higher than the baseline glucose indicated glucose resistance in the HFD-fed mice. **E** Insulin levels during IPGTT. Results are mean ± SEM. ^***^ P < 0.001, *n* = 50. **F** Glucose levels during ITT. Results are mean ± SEM. ^*^ P < 0.05, ^***^ P < 0.001. *n* = 50. **G** Glucose area under the curve during ITT. After correction, the blood glucose level is multiplied -1. Results are mean ± SEM. ^***^ P < 0.001. *n* = 50. **H** Representative Evans blue and TTC stained sections after MI/R injury. **I** Infarct/area at risk% (IA/AAR%). *n* = 6, Results are mean ± SEM. ^**^ P < 0.01 versus ND + MI/R group. **J** The expression of 8-OHdG

All animal experiments were approved by the Animal Care and Use Committee of Renmin Hospital of Wuhan University and were performed in compliance with the *Guidelines for Care and Use of Laboratory Animals* published by the US National Institutes of Health (NIH Publication No. 85–23, revised 1996). All efforts were aimed at minimising animal suffering and reducing the number of animals used, according to the generally accepted “3Rs” (Replacement, Reduction, and Refinement). Male C57BL/6 mice (8-week-old) were purchased from Beijing Vital River Laboratory Animal Technology (Beijing, China) and kept in a clean isolation cage with free access to a standard laboratory chow diet. T2D was induced by high-fat diet (HFD; a diet incorporating 60% fat calories) and low-dose STZ (90 mg/kg) [18]. Briefly, mice were first acclimated for a week, fed HDF for one month, and then induced by low-dose STZ. The intraperitoneal glucose tolerance test (2 mg/kg body weight; IPGTT) and insulin resistance test (0.75 U insulin/kg body weight; ITT) were started 4 weeks before surgery to ensure the success of the model (Fig. 1 A-G). A model which incorporates an HFD to induce peripheral insulin resistance, followed by STZ to target pancreatic β-cells, would closely mimic not only the phenotype but also the pathogenesis of humans. The control group was fed a normal caloric diet (ND) and injected with 0.01 M sodium citrate buffer (pH 4.5).

The surgical area was then prepared and disinfected, mice were anesthetized with isoflurane. The skin was cut at the 3rd to 4th intercostal space on the left side of the sternum. The subcutaneous tissue and muscle were bluntly separated layer-by-layer. The chest cavity was opened, the heart exposed, and the pericardium cut. The anterior descending branch (LAD) of the left coronary artery (LAD) is a pink blood vessel that appears on the lower or left side of the heart. A 6–0 suture needle was used to pass through the myocardium 1 mm from the lower border of the atrial appendage, and the LAD was ligated. Before ligation, a 0.2–0.3 cm 10-gauge polyethylene tube was placed under the line. After ligation of the LAD, the anterior wall of the left ventricle was found to be white, the local systolic movement was limited, the left atrial appendage was full, and the ECG showed continuous elevation of the ST segment, indicating successful myocardial ischaemia. The thoracic cavity was closed by suture; the thoracic cavity was opened again after 30 min of ischaemia, the polyethylene tube was removed, and the ligature was cut. The heart surface changed from pale to red, and the elevated ST segment of the electrocardiogram fell back. Reperfusion was judged to be successful and it was continued for 2 h.

To understand the role of mitochondrial fusion in diabetic myocardial ischaemia–reperfusion injury, we constructed recombinant adeno-associated viruses (rAAV) rAAV-MFN2 and rAAV-OPA1. The mice were randomised into the following groups (*N* = 6–8): HDF + STZ + sham operation (sham group), HDF + STZ + MI/R (MI/R group), MI/R + rAAV-OPA1 (rAAv-OPA1 group), and MI/R + rAAV-MFN2 (rAAv-MFN2 group). rAAV injection of the heart was performed 3 weeks in advance to build an overexpression model. Specifically, 5 × 10^11^ VG/mL of virus was injected at each point around the ventricular wall.

To investigate the role of H-151 in functional and structural changes induced by I/R injury in diabetes, three groups of male mice were randomly divided into (1) HFD + STZ + H-151 group (H-151 group), (2) HFD + STZ + MI/R + vehicle group (MI/R + vehicle), and (3) HFD + STZ + MI/R + H-151 group (MI/R + H-151 group) (*N* = 6–8). H-151 treated mice were injected intraperitoneally with 750 nmol per mouse in 200 µl 10% Tween-80 in PBS, as previously described [19]. Mice in the vehicle control group were injected intraperitoneally with 200 µl saline.

To determine the therapeutic effects of CoQ10, mice were randomly divided into three groups (*N* = 6–8): (1) HFD + STZ + CoQ10 group (CoQ10 group), (2) HFD + STZ + MI/R + vehicle group (MI/R + vehicle group), and (3) HFD + STZ + MI/R + CoQ10 group (MI/R + CoQ10 group). CoQ10 was dissolved in soybean lecithin and administered to mice intraperitoneally after ischaemic treatment at a dose of 5 mg/kg, as previously described [20]. Isovolumetric soybean lecithin was injected in the MI/R + vehicle group.

### Intraperitoneal glucose tolerance test (IPGTT), Insulin tolerance test (ITT)

The intraperitoneal glucose tolerance test (2 mg/kg body weight; IPGTT) and insulin resistance test (0.75 U insulin/kg body weight; (ITT) were started 4 weeks before surgery to ensure the success of the model. The test method was previously described [21].

### Echocardiography

The details of transthoracic echocardiography were described in our previous study [22]. In brief, baseline echocardiography was performed in mice three days before MI/R to detect cardiac function. MI/R echocardiography was performed immediately after suturing the parasternal skin. Parasternal long-axis and short-axis images were obtained in two-dimensional and M-mode for quantification. Left ventricular internal dimension systole (LVIDs) and left ventricular internal dimension diastole (LVIDd) were measured in the parasternal LV long-axis view. The left ventricular ejection fraction (LVEF) and left ventricular shortening fraction (LVFS) were calculated using computer algorithms.

### Measurement of infarct size

Details of measurement of infarct size have been described in our previous study [22]. In short, myocardial infarct size was measured using 0.3% Evans Blue dye and 1% TTC at the end of reperfusion. The myocardial area at risk (AAR) and infarct size were detected using a scanner (Epson, v30, Japan), and data were analysed using ImageJ software. The blue area is the normal myocardium, red indicates ischaemic myocardium, pale denotes myocardial infarction, and the infarct size and percentage of AAR were calculated.

### Immunohistochemistry

After reperfusion, tissues from the apical heart region were collected, fixed in 4% buffered paraformaldehyde, and embedded in paraffin. The sections were incubated with antibody (1:200) overnight at 4 °C. After elution, the samples were stained using a histochemical kit, sealed with neutral gum, and air-dried. The sections were stained with HE eosin to assess the heart morphology and myocardial fibre integrity. Images were captured by an OLYMPUS (BX53) microscope.

### Transmission electron microscopy

After reperfusion, tissues from the apical heart region were immediately collected, dissected into 1–2 mm^3^ pieces, and fixed with 2.5% glutaraldehyde and 0.1 M sodium cacodylate buffer. The collected samples were embedded, subjected to ultramicrotomy to obtain 90-nm thin sections, and images were captured using a Hitachi HT7800 120 kv transmission electron microscope (Hitachi, Japan).

### Lactate dehydrogenase (LDH) activity, troponin T(cTnT), creatine kinase (CK)-MB, and cell viability

After reperfusion or special treatment, blood samples or culture medium supernatants from the heart or cells were collected immediately, left standing, centrifuged at 8000 × g at 4 ℃ for 5 min, and the supernatant was collected for inspection of LDH, cTnT, and CK-MB. Cell viability was analysed using a cell counting kit-8 (ServiceBio, Wuhan, Hubei, China).

### Tissue mitochondria isolation

Fresh infracted area tissue was quickly weighed, washed once with pre-cooled PBS, and cut into 3 mm2 tissue fragments on ice. Ten times the volume of pre-cooled mitochondrial lysis buffer was added (to obtain cytoplasmic protein, PMSF was added in advance until the PMSF concentration was 1 ×), placed in a homogeniser in an ice bath, and homogenised 15 times, and centrifuged at 2000 × g for 3 min at 4 °C to remove nuclei, unbroken cells, and large membrane debris. The supernatant was transferred to a clean centrifuge tube and centrifuged at 6000 × g for 10 min at 4 °C and precipitated as mitochondria. To obtain mitochondria-depleted cytoplasmic proteins, the supernatant was collected at this step, taking care not to touch the pellet when collecting the supernatant. The collected supernatant was then centrifuged at 12,000 × g at 4 °C for 10 min, the supernatant contained the cytoplasmic protein.

### Cell mitochondria isolation

The cells were washed once with pre-cooled PBS, centrifuged at 1000 × g for 5 min at 4 °C and the supernatant was discarded. The cells were resuspended in 1–2 ml of pre-cooled mitochondrial lysis buffer and placed in an ice bath for 10–15 min. The cell suspension was transferred to a homogeniser and homogenised 10–20 times. The homogenate was immediately removed, an equal amount of wash buffer was added, and the mixture was gently inverted several times. The mixture was centrifuged at 1300 × g for 5 min at 4 °C to remove nuclei, unbroken cells, and large membrane debris. The supernatant was transferred to a clean centrifuge tube and centrifuged at 1000 × g for 5 min at 4 °C; this was repeated twice. The supernatant was transferred to a clean centrifuge tube and centrifuged at 12,000 × g for 15 min at 4 °C and pellet as mitochondria.

### Isolation and detection of cytosolic mtDNA by qRT-PCR

All samples were centrifuged at 700 × g at 4 °C for 10 min to remove the nuclei and intact cells. The supernatant was normalized to the same volume according to the protein concentration. To isolate the cytosolic fraction, the supernatant was further centrifuged at 10,000 × g for 30 min at 4 °C. Quantitative PCR was used to detect the levels of cytosolic mitoDNA, and the sequences that coded for mitoDNA were used as primers. The primer sequences are shown in Supplementary Table 1. The data were analyzed using the 2^−ΔΔCt^ method and normalized to GAPDH.

### Quantitation of mitoDNA in serum

After the intervention, the thoraxes were opened rapidly and blood samples were collected by heart puncture and centrifuged at 3000 rpm for 10 min to separate the serum from cellular blood components. The serum was stored at − 80 °C for later mitoDNA and cytokines assay. mitoDNA abundance was measured by qRT-PCR. The primer sequences are shown in Supplementary Table 1. The data were analyzed using the 2^−ΔΔCt^ method and normalized to GAPDH.

### ATP content, Malondialdehyde (MDA) content, Complex I, II, V enzyme activity

The levels of ATP, malondialdehyde (MDA), and Complex I, II, and V enzyme activity were measured according to the manufacturer’s instructions. The kits used were as follows: ATP Microplate Assay Kit (abs580117), General Malondialdehyde (MDA) ELISA Kit (MBS2000071, MyBioSource), Complex I enzyme activity microplate assay kit (ab109721, Abcam), Complex II enzyme activity microplate assay kit (ab109908, Abcam), and MitoTox™ complex V OXPHOS activity assay kit (ab109907, Abcam).

### Neonatal mouse ventricular myocyte (NRVM) isolation and H9C2 cells culture

Neonatal mouse ventricular myocytes (NMVMs) were isolated from the hearts of neonatal C57BL/6 mice (1–3 days). The suckling mice were put into 75% ethanol for disinfection. The chest was opened and the heart removed, then placed in a dish containing D-Hanks. The residual blood was removed, and the heart was minced into 1–3 mm^3^ pieces and digested using digestion buffer (0.08% trypsin buffer and 0.05% collagenase II) at 37 ℃ in a water bath for 5 min. After discarding the supernatant, the heart tissue was re-digested in a water bath for 20 min and shaken every 2 min. Then, cold 10% DMEM/F12 was added to stop the digestion; the slurry was centrifuged at 12,000 × g for 5 min and the supernatant was discarded. The cells were seeded into 10 cm^2^ culture dishes and maintained in a culture hub with 95% air and 5% CO_2_ at 37 ℃ for 2 h to make fibroblasts preferentially adhere to the bottom of the culture flasks. The supernatant containing unattached cardiomyocytes was transferred to a new cell container for further experiments. H9C2 cells were cultured in 10% DMEM in the same culture hub as the NMVMs. To simulate an HDF and high-glucose (HG) model on cells using PA (800 µM) and glucose (25 mM), mannitol (25 mM) was used to control osmotic effects at high glucose concentrations. The hypoxia-reoxygenation (H/R) model was simulated by hypoxia for 4 h (94% N_2_ + 5% CO_2_ + 1% O_2_) and reoxygenation (95% air + 5% CO_2_) for 2 h followed by shRNA transfection using lipo8000™ (Beyotime, Shanghai, China).

### Oxygen consumption rate (OCR)

The mitochondrial respiratory capacity was determined by measuring the oxygen consumption rate (OCR) in real time using a Seahorse XFe 96 extracellular flux analyser (Seahorse Biosciences, Agilent Technologies, USA). Briefly, NMVMs were seeded in an assay microplate at 5 × 10^3^ cells/well in 10% DMEM/F12. After specific stimulation, the culture medium was replaced with Seahorse XF DMEM buffer (containing 10 mM glucose, 2 mM glutamine, and 1 mM pyruvate) and the plate was incubated at 37 ℃ for 1 h. The plate was then sequentially injected with the following compounds: oligomycin 1.5 mM, FCCP 3 mM, Rotenone 0.5 mM (101,706–100, XF Cell Mito Stress Test Kit). Respiratory parameters (OCR), including basal respiration, ATP production-coupled respiration, maximal respiration, and spare respiratory capacity, were quantified in real time by calculating the average respiratory rate and subtracting the rates before and after the compound injection. The OCR in each microplate well was normalised to the protein content in each well.

### MitoTracker and LysoTracker

After special treatment, the cells were labelled with mitochondria and lysosomes, which were tracked using commercial MitoTracker Red CMXRos (C1049B) and LysoTracker Green (C1047S), according to the manufacturer’s instructions.

### RNA extraction and quantitative real-time PCR (qRT-PCR)

Total RNA was extracted from tissues and cultured cells according to standard protocols using TRIzol reagent, and 1 ug of total RNA was reverse transcribed into cDNA using a One-Step gDNA Remover kit (G3337, ServiceBio, Wuhan, Hubei, China) with a gDNA eraser. cDNA (0.5 µl) was then mixed with Universal Blue SYBR Green qPCR Master Mix (G3326, ServiceBio, Wuhan, Hubei, China) and 0.25 µM primers to obtain a final volume of 10 µl, which was subjected to quantitative real-time PCR using a Bio-Rad CFX Connect System (Bio-Rad, Hercules, California, U.S.). The relative mRNA expression of specific genes was quantified using the comparative 2^−∆∆Ct^ method and normalised to that of GAPDH. Primers used for each gene are listed in Supplementary Table 1.

### Immunofluorescence staining

After specific treatments, the cells were fixed with pre-warmed 4% formaldehyde buffer at 37 °C for 30 min, washed three times with PBS, then permeabilised with 5% Triton X-100 for 10 min at room temperature washed three times with PBS, then blocked with 1% BSA for 30 min. After incubation with STING (1:50) at 4 °C overnight and FITC conjugated Goat Anti-Mouse IgG (GB22301, ServiceBio, Wuhan, Hubei, China) at 24 ℃ for 1 h, nuclei were stained with DAPI and an anti-fluorescence quencher before mounting on a slide for observation.

### Western blotting (WB)

After the intervention, the total cell proteins and myocardial tissues were extracted. The bicinchoninic acid method was used to determine protein concentration and quantify the total amount of protein. Sodium dodecyl sulphate–polyacrylamide gel electrophoresis was performed to separate the protein extracts. After electrophoresis, the gel was transferred onto a polyvinylidene fluoride (PVDF) membrane. The membranes were incubated with 5% skim milk for 1 h. Primary antibodies were then added to incubate with the membranes at 4 ℃ overnight. HRP-conjugated secondary antibody was incubated for 1 h at room temperature. Blotting was detected using an ECL detection reagent (ChemiDoc, BIO-RAD). The antibodies and their dilutions used in this study are listed as follows: MFN2 (#11925, 1:1,000), OPA1 (#80471, 1:1,000), DRP1 (#8570, 1:1,000), cGAS (PA5-121188, 1:1,000), STING (#50494, 1:1,000), p-TBK1 (Ser172) (#5483, 1:1,000), TBK1 (#38066, 1:1,000), p-IRF3 (Ser396) (#29047, 1:1,000), IRF3 (SD2062, 1:1,000), PINK1 (PA1-16604, 1:1,000), Parkin (#4211, 1:1,000), P62 (#23214, 1:1,000), LC3 II (#2775, 1:1,000), Cyto c (#4280, 1:1,000).

### Statistical analyses

All data are presented as the mean ± SEM and were analysed using GraphPad Prism 9 software. Normal distribution of data was assessed using the Shapiro–Wilk normality test. Differences between two groups were analysed using the Student’s unpaired t-test. Multiple comparisons among different groups were analysed using one-way ANOVA followed by a post hoc Tukey’s test. Statistical significance was defined as *P* < 0.05, *P* < 0.01, *P* < 0.001, and *P* < 0.0001.

## Results

### Escape of mitochondrial DNA (mitoDNA) into the cytosol and cGAS-STING signalling activation in the hearts of diabetic MI/R mice

**Fig. 2:**
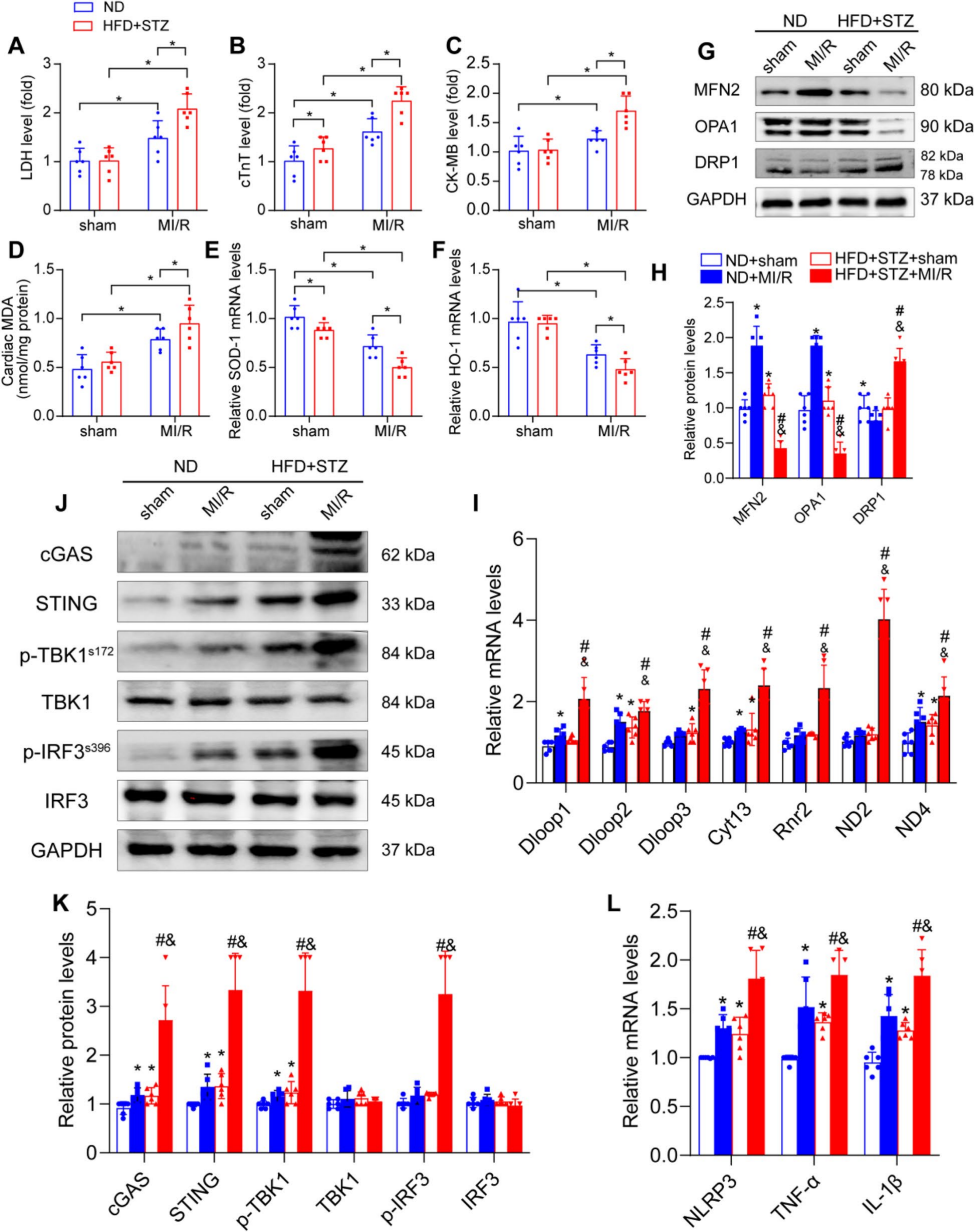
Diabetic MI/R injury aggravated, mitochondrial dynamic imbalance and mitoDNA leakage, activation of cGAS-STING pathway and inflammation. The mice were subjected to 120 min of reperfusion after 30 min of ischaemia. **A**-**C** LDH, cTnT, and CK-MB activities in the serum. **D** MDA content in the myocardial tissue. **E**–**F** mRNA levels of SOD-1 and HO-1 in the myocardial tissue. All data are presented as mean ± SEM from six independent experiments, *n* = 6 mice per group. ^*^P < 0.05. **G** Mitochondrial fusion and fission protein contents in myocardial tissue were detected by western blotting. **H** Quantification of G. **I** The mRNA levels of Dloop1, Dloop2, Dloop3, CytB, Rnr2, ND2, and ND4 in myocardial tissue were detected by qRT-PCR. **J** Representative protein levels of cGAS, STING, p-TBK1^s172^, TBK1, p-IRF3^s396^ and IRF3 detected by western blotting. **K** Quantification of J. **L** mRNA levels of NLRP3, TNF-α, and IL-1β detected by qRT-PCR. All data are presented as mean ± SEM, *n* = 6 mice per group. ^*^P < 0.05 versus ND + sham group; ^#^*P* < 0.05 versus ND + MI/R group; ^&^*P* < 0.05 versus HFD + STZ + sham group

Increased myocardial vulnerability in diabetes has been reported in many previous studies including by us [6, 23]. As expected, diabetic MI/R induced severe myocardial injury in mice, as verified by the increased serum levels of LDH, cTnT, and CK-MB, which all decreased in mice with non-diabetic MI/R (Fig. 2 A-C), accompanied by an increased myocardial infarct area, stained with Evans blue and TTC (Fig. 1 H-I). Mitochondria are especially abundant in cardiac tissues; hence, mitochondrial dysregulation and reactive oxygen species (ROS) production are thought to contribute significantly to cardiac pathology. We found that cardiac MDA was increased in diabetic MI/R injury, which was accompanied by decreased mRNA levels of antioxidant markers (SOD-1 and HO-1) (Fig. 2 D-F). Next, we found that diabetic MI/R injury decreased MFN2 and OPA1 and increased DRP1, suggesting that mitochondrial dynamics change in diabetic MI/R injury with less fusion and more fission (Fig. 2 G-H). Cytoplasmic mitoDNA can be converted to the second messenger cGAMP, which activates the DNA sensor cGAS-STING signalling, leading to the transcription of pro-inflammatory molecules and activation of the inflammatory response [24, 25]. Hence, we detected the levels of mitoDNA (Dloop1, Dloop2, Dloop3, CytB, Rnr2, ND2, and ND4) in the cytosol and found that all of these mitoDNAs were upregulated in the cytosol of diabetic MI/R mice compared with the non-diabetic MI/R injury group (Fig. 2 I), indicating leakage of mitoDNA into the cytosol. The circulating cell-free mitoDNA in the diabetic MI/R injury group were found to be upregulated compared with the non-diabetic MI/R injury group (Supplemental 1 A). Immunohistochemistry also confirmed increased damage to DNA (Fig. 1 J).

The cGAS-STING signalling pathway, comprising the synthase for the second messenger cyclic GMP-AMP (cGAS) and cyclic GMP-AMP receptor stimulator of interferon genes (STING), detects pathogenic DNA to trigger an innate immune reaction involving a strong type I interferon response. In view of the high levels of cytoplasmic mitoDNA in diabetic MI/R mice, we next detected levels of the cGAS-STING pathway and inflammation-stimulated factors (ISFs: NLRP3, TNF-α, and IL-1β) and found that the cGAS-STING signalling pathway was activated in diabetic MI/R mice (Fig. 2 J–K). Furthermore, the mRNA levels of inflammation-stimulated factors were elevated (Fig. 2 L). These results demonstrate that disturbances of mitochondrial dynamic may lead to the escape of mitoDNA into the cytosol, accompanied by activation of the cGAS-STING signaling pathway, thereby eliciting an inflammatory response in diabetic MI/R injury mice.

### Overexpression of MFN2 significantly regulated mitochondrial dynamics and reduced mitoDNA spillover into the cytoplasm in diabetic MI/R mice

**Fig. 3:**
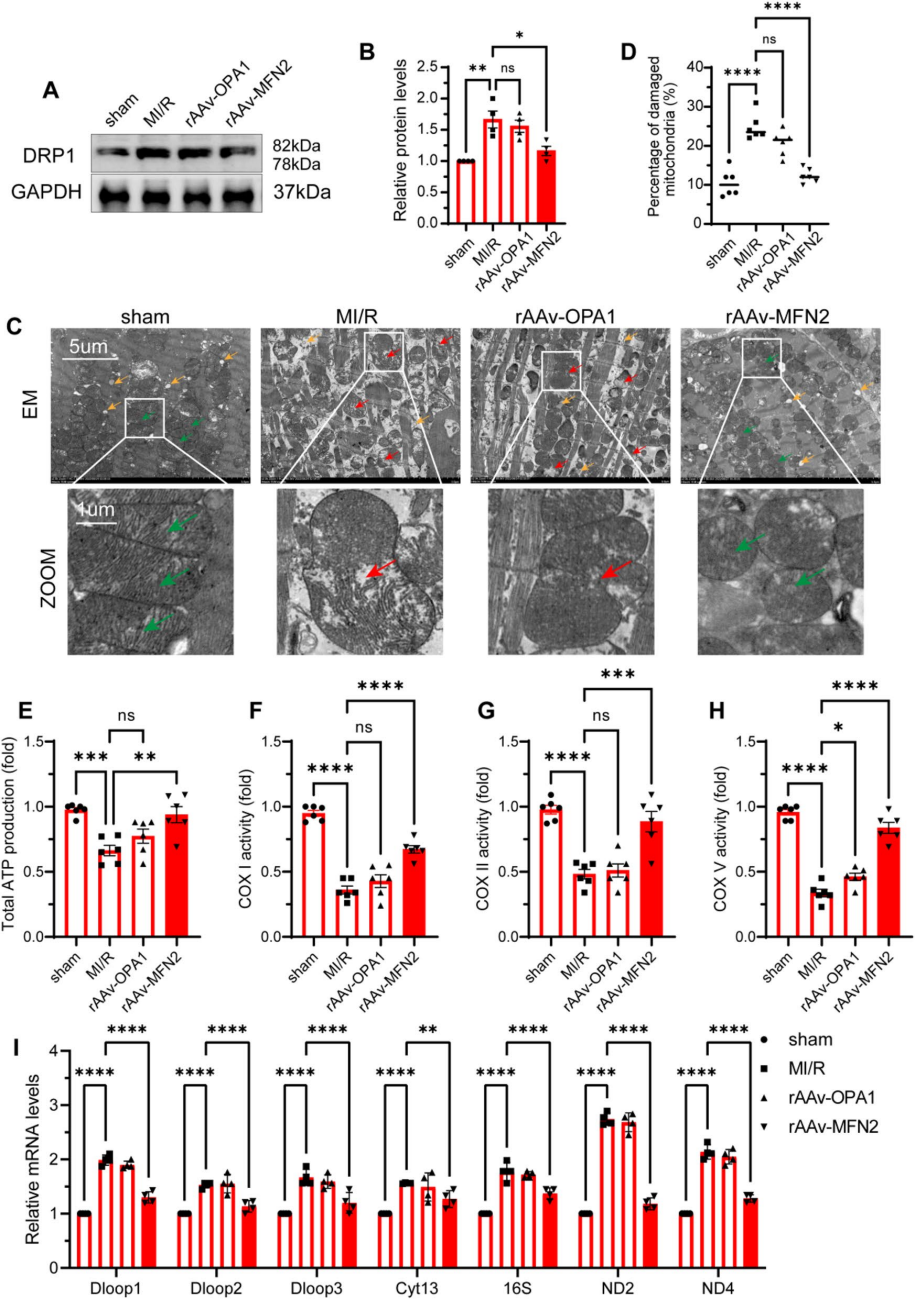
Overexpression of rAAV-MFN2 regulated mitochondrial dynamics and function in diabetic MI/R mice. Three weeks before the HFD + STZ mice were stabilised;saline, rAAV-OPA1 5 × 10 ^11^ VG/mL and rAAV-MFN2 5 × 10 ^11^ VG/mL were injected via tail vein injection. After 3 weeks of transfection, the normal diet and HFD + STZ mice were subjected to 120 min of reperfusion after 30 min of ischaemia. **A** Protein levels of DRP1 were detected using western blotting. **B** Quantification of A. Data are presented as the mean ± SEM. ^*^P < 0.05, ^**^P < 0.01. **C**-**D** Representative EM images of mitochondrial morphology. Data are shown as a dot plot of the percentage of damaged mitochondria from six images in each group, ^****^P < 0.0001. Scale bar: 5 µm. Zoom scale bar: 1 µm. Yellow arrow: lipid droplets. Red arrow: damaged mitochondria. Green arrow: normal mitochondria. **E**–**H** Total ATP production and ETC complex activity in cardiomyocytes. All data are presented as the mean ± SEM. ^*^P < 0.05, ^**^P < 0.01, ^***^P < 0.001, ^****^P < 0.0001. **I** The mRNA levels of Dloop1, Dloop2, Dloop3, CytB, Rnr2, ND2, and ND4 in the myocardial tissue were detected by qRT-PCR. Data are presented as the mean ± SEM. ^*^P < 0.05, ^**^P < 0.01, ^***^P < 0.001, ^****^P < 0.0001

The dynamic processes of mitochondrial fission and fusion are tightly regulated, determine the mitochondrial shape, and influence mitochondrial function. To elucidate the role of mitochondrial fusion in the regulation of diabetic MI/R injury, we explored the effects of rAAv-OPA1 and rAAv-MFN2 overexpression in diabetic MI/R mice (Supplemental 2 A-B). We found that rAAv-MFN2, but not rAAv-OPA1, was a prerequisite for the adaptation of mitochondrial dynamics. Immunoblot analyses revealed that r-AAv-MFN2 decreased the level of DRP1, indicating mitochondrial dynamic changes in diabetic MI/R with more fusion and less fission (Fig. 3 A–B). An electron microscope (EM) was used to assess the architecture of the myocardium. We found that more mitochondria contained disorganised cristae and more swollen mitochondria in the MI/R group than in the sham group, and these changes mostly disappeared in the rAAv-MFN2 group, but not in the rAAv-OPA1 group (Fig. 3 C,D). Mitochondrial function is characterised by mitochondrial ATP production and the activity of mitochondrial electron transport chain (ETC) complexes (COX I, COX II, and COX V). As expected, overexpression MFN2 significantly increased mitochondrial ATP production and activated COX I, COX II, and COX V (Fig. 3. E–H) in the HFD + STZ-induced diabetic MI/R hearts compared to the MI/R group. Moreover, our investigation showed that the levels of cytosolic mitoDNA decreased in diabetic MI/R mice after rAAv-MFN2 treatment (Fig. 3 I). These data indicate that transfection of rAAv-MFN2, but not rAAv-OPA1, regulated mitochondrial dynamics and function and reduced mitoDNA spillover into the cytoplasm in diabetic MI/R mice.

Mitophagy, the selective removal of damaged mitochondria, is a fundamental process critical for mitochondrial quality control. PINK1/Parkin is a canonical pathway that mediates mitophagy. We have previously shown that mitochondria-targeted antioxidant MitoQ ameliorates MI/R injury by enhancing PINK1/Parkin-mediated mitophagy in type 2 diabetic rats [26]. In our study, we found that overexpression of the mitochondrial fusion protein MFN2 in diabetic MI/R mice restored PINK1/Parkin mediated mitophagy, and this was confirmed by increased expression of PINK1, Parkin, LC3 II, and decreased P62 (Fig. Supplemental 2 C,D). This suggests that the restoration of mitochondrial fusion may have an activating effect on mitophagy.

### Overexpression of MFN2 reversed TBK1-IRF3 pathway phosphorylation, inhibited inflammatory response, and improved cardiac function in diabetic MI/R mice

**Fig. 4:**
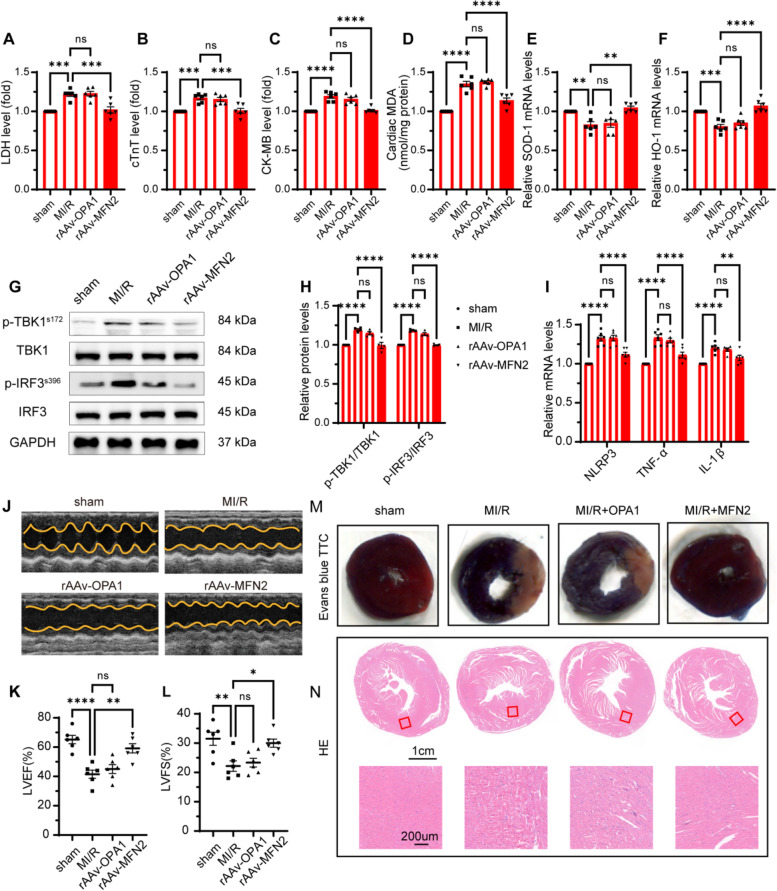
Overexpression of rAAV-MFN2 alleviated myocardial oxidative damage, inhibited phosph-TBK1/IRF3 pathway activity, and reduced inflammatory response in diabetic MI/R mice. **A**-**C** LDH, cTnT and CK-MB activity in serum. **D** MDA content in myocardial tissue. **E**–**F** mRNA level of SOD-1 and HO-1 in myocardial tissue. All data are presented as mean ± SEM, *n* = 6 mice per group. ^**^P < 0.01, ^***^P < 0.001, ^****^P < 0.0001. **G** Representative protein levels of p-TBK1^s172^, TBK1, p-IRF3^s396^ and IRF3 were detected by western blotting. **H** Quantification of G. Data are presented as mean ± SEM. ^****^P < 0.0001. **I** mRNA levels of NLRP3, TNF-α, and IL-1β were detected by qRT-PCR. All data are presented as mean ± SEM. ^**^P < 0.01, ^****^P < 0.0001. **J** Left ventricular ejection fraction (LVEF, *n* = 6). **K** Left ventricular fractional shortening (LVFS, *n* = 6). All data are presented as mean ± SEM. ^*^P < 0.05, ^**^P < 0.01, ^****^P < 0.0001. **L** Representative images of Evans blue and TTC stained heart sections after MI/R or sham operation. **M** Representative images of HE-stained heart sections after MI/R or sham operation

We examined whether restoration of mitochondrial fusion could regulate cardiac function in diabetic MI/R injury. Under sham conditions, LDH, cTnT, and CK-MB production were at low levels; following MI/R stimulation these levels were high and could be reversed by rAAV-MFN2 transfection (Fig. 4 A–C). Compared with the MI/R group, the rAAv-MFN2 group had enhanced anti-oxidative damage, which was manifested by a decrease in MDA and an increase in SOD-1 and HO-1 (Fig. 4 D–F). Next, we found that restoration of mitochondrial fusion can inhibit the activity of phospho-TBK1 and phospho-IRF3 and reduce cardiac inflammation in HFD-fed STZ-induced diabetic mice (Fig. 4 G–I). Echocardiographic analysis revealed that rAAv-MFN2 administration detrimentally affected the exerted protective effects in MI/R mice, as evidenced by a significant increase in the left ventricular ejection fraction and left ventricular fraction shortening compared with MI/R group mice (Fig. 4 J-L). Histological analysis showed that rAAv-MFN2 rescued myocardial fibre integrity in diabetic MI/R mice compared to that in the MI/R group mice (Fig. 4 N). Furthermore, Evans-TTC staining showed that rAAv-MFN2 rescued the myocardial infarction size in diabetic MI/R mice compared to that in the MI/R group mice (Fig. 4 M). These data suggested that promoted mitochondrial fusion reduced oxidative stress damage and inflammatory response in the diabetic myocardium, thereby improving MI/R injury heart function.

### Palmitic acid drives cardiomyocyte mitochondrial dysfunction in vitro

To determine whether MI/R in diabetes modulated cardiomyocyte mitochondrial dysfunction in vitro, H9C2 cells were cultured in both high glucose HG (25 mM) and palmitate (incubated with different concentrations of PA 0, 200, 400, 800, and 1600 µM) (Fig. Supplemental 3 A). Palmitic acid is the most prevalent saturated free fatty acid in humans and has a high hazard ratio for diabetes development [27], and was used to mimic lipid overload in H9C2 cardiomyocytes. We found that the levels of ATP in the supernatant were upregulated in a dose-dependent manner by PA (Fig. Supplemental 3 B); however, the intracellular ATP peaked at 400 µM and then started to downregulate (PA concentration was 800 µM) (Fig. Supplemental 3 C). Consistent with the ATP results, downregulation of mitochondrial respiratory ETC complexes was observed at 800 µmol/L. (Fig. Supplemental 3 D-F). Next, we assessed the protein levels of mitochondrial and cytoplasmic cytochrome C (which plays a vital role in mitochondrial ETC and Cyto C) at different PA concentrations. We found that more Cyto C was released from the mitochondria into the cytoplasm after 800 µmol/L PA treatment than in the low concentration groups, suggesting that mitochondrial damage was aggravated (Fig. Supplemental 3 G,H). Taken together, these data suggest that mitochondrial function begins to be impaired at 800 µM PA; therefore, 800 µM PA was chosen as the final concentration for intervention, allowing us to explore the direct effects of mitochondrial function in vitro.

### HG + PA + HR aggravated cardiomyocyte injury and exacerbated cytoplasmic mitoDNA accumulation and inflammation

**Fig. 5:**
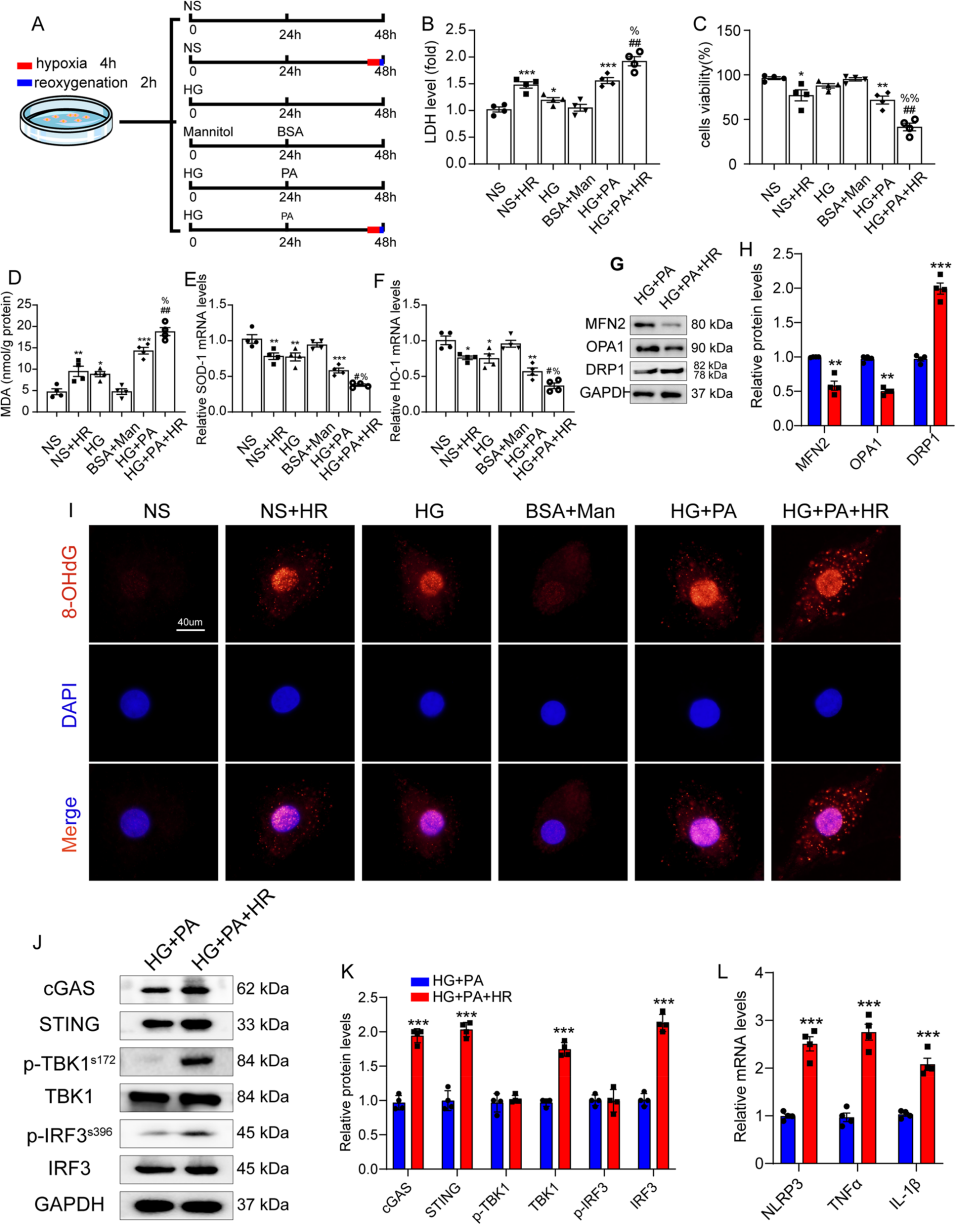
HG + PA + HR aggravated cardiomyocyte injury and exacerbated cytoplasmic mitoDNA accumulation and inflammation. **A** Schematic diagram of H9C2 cell experiment, NS (normal condition: low glucose 5 mM), HG (high glucose: 25 mM), PA (palmitate: 800 µM). **B**-**F** The LDH activity, cells viability, MDA content, SOD-1 and HO-1 mRNA levels were detected. Data are presented as mean ± SEM. ^*^P < 0.05, ^**^P < 0.01 and ^***^P < 0.001 vs. NS group; ^#^*P* < 0.05 and ^##^*P* < 0.01 vs. NS + HR group; ^%^*P* < 0.05 and ^%%^*P* < 0.01 vs. HG + PA group. **G** Representative protein levels of MFN2, OPA1 and DRP1 were detected by western blotting. **H** Quantification of G. Data are presented as mean ± SEM. ^**^P < 0.01, ^***^P < 0.001. **I** Representative fluorescent images of 8-OHdG. Scale bar: 40 μm. **J** Representative protein levels of cGAS, STING, p-TBK1^s172^, TBK1, p-IRF3^s396^ and IRF3 were detected by western blotting. **K** Quantification of J. **L** The mRNA levels of NLRP3, TNF-α, and IL-1β were detected by qRT-PCR. Data are presented as mean ± SEM. ^***^P < 0.001

Our experimental results showed that high glucose and palmitic acid aggravated hypoxia-reoxygenation injury in H9C2 cardiomyocytes, manifested as increased LDH release and decreased cell viability compared to other groups (Fig. 5 B,C). Next, we examined MDA activation and mRNA levels of antioxidant markers and found that MDA increased in the HG + PA + HR group, which was accompanied by decreased mRNA levels of SOD-1 and HO-1 (Fig. 5 D–F). We showed that diabetic MI/R affects mitochondrial fusion and fission in vivo. Consistently, we also observed that HG + PA + HR in H9C2 cells reduce mitochondrial fusion and increased mitochondrial fission compared to the HG + PA group (Fig. 5 G,H). Furthermore, we examined cytoplasmic mitoDNA levels using qRT-PCR and found that levels of cytosolic mitoDNA increased after PA and HR treatment compared to the HG + PA group (Fig. Supplemental 4 A), accompanied by increased DNA oxidative damage, as shown by 8-OHdG staining (Fig. 5 I). We next detected the levels of cGAS-STING pathway and inflammation-stimulated factor expression and found that the cGAS-STING signalling pathway was extremely activated in the HG + PA + HR group (Fig. 5 J,K) and the mRNA levels of inflammation-stimulated factors were elevated in the HG + PA + HR group (Fig. 5 L). These data indicated that glucolipotoxicity dramatically aggravated cardiomyocyte injury and mitochondrial oxidative injury, and may trigger activation of the cGAS-STING signalling pathway, resulting in an inflammatory response in H9C2 cells with hypoxia and reoxygenation. This finding is consistent with our in vivo findings.

### EtBr rescued shOPA1 or shMFN2-induced mitochondrial DNA extravasation

**Fig. 6:**
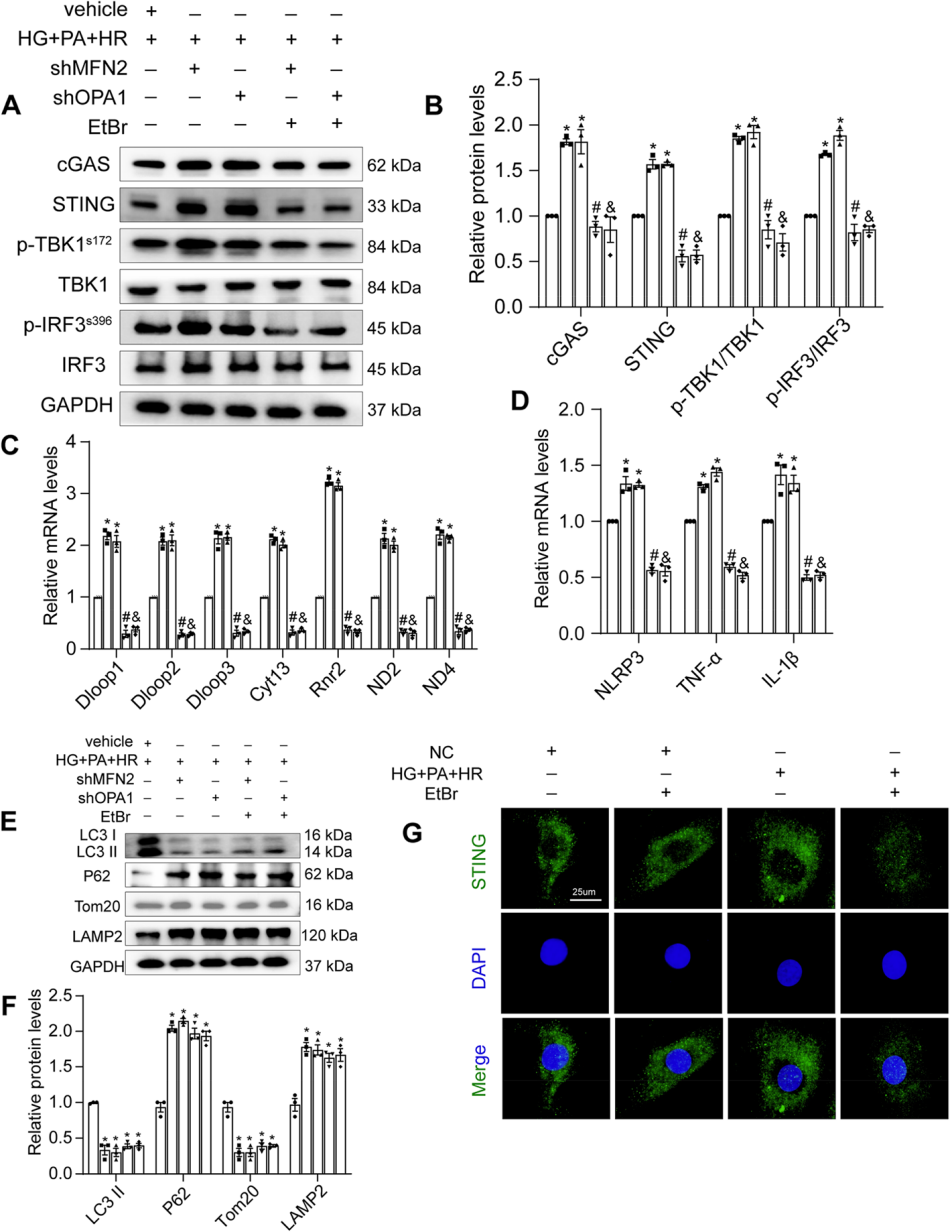
EtBr rescued shOPA1 or shMFN2-induced mitochondrial DNA extravasation. **A** Representative protein levels of cGAS, STING, p-TBK1^s172^, TBK1, p-IRF3^s396^ and IRF3 were detected by western blotting. **B** Quantification of A. **C** The mRNA levels of Dloop1, Dloop2, Dloop3, CytB, Rnr2, ND2, and ND4 in H9C2 cells were detected by qRT-PCR. **D** The mRNA levels of NLRP3, TNF-α, and IL-1β were detected by qRT-PCR. **E** Representative protein levels of LC3-II, P62, Tom20, and LAMP2 were detected by western blotting. **F** Quantification of E. Data are presented as mean ± SEM. ^*^P < 0.05 vs. vehicle group, ^#^*P* < 0.05 vs. shMFN2 group, ^&^*P* < 0.05 vs. shOPA1 group. **G** Representative fluorescent images of STING. Scale bar: 25 μm

To further confirm the effect of mitochondrial fusion and fission on the cGAS-STING pathway and inflammatory response, we silenced OPA1 or MFN2 by transfecting short hairpin RNAs (shRNAs) (Fig. Supplemental 5 A,B), and treating with ethidium bromide (EtBr). Chronic EtBr exposure results in reduction of mitoDNA [28]. We found that shOPA1 and shMFN2 caused severe ultrastructural defects in the mitochondria, including swollen mitochondria and defects in cristae organisation, such as concentric inner membranes (Fig. Supplemental 5 C). We found that compared with the vehicle group, the cGAS-STING pathway was significantly activated in the shOPA1 and shMFN2 groups, which could be reversed by EtBr (Fig. 6 A,B). Next, we detected the expression of cytoplasmic mitoDNA and found that the cytoplasmic expression of mitoDNA in the shOPA1 and shMFN2 groups was significantly upregulated and could be reversed by EtBr, as shown by mitoDNA levels using qRT-PCR (Fig. 6 C). Finally, we detected the inflammation of the cells and found that the inflammation was significantly aggravated in the shOPA1 and shMFN2 groups, and EtBr reduced the inflammatory response (Fig. 6 D). Taken together, the above experiments demonstrated that mitoDNA in cardiomyocytes is a key pro-inflammatory mediator, and its release is closely related to the homeostasis of mitochondrial fusion.

Previous data have shown that disturbances in mitochondrial dynamics are not only alterations in mitochondrial fission and fusion but also in mitophagy. Interestingly, we found that EtBr did not ameliorate the inhibition of mitochondrial fusion protein-induced autophagy, which was manifested by a marked increase in P62 and LAMP2 expression and downregulation of LC3II and Tom20 (Fig. 6 E,F). With further co-staining by MitoTracker, a mitochondrial marker, we hardly observed colocalised LC3II and MitoTracker in the shOPA1 and shMFN2 groups, and this could not be reversed by EtBr (Fig. Supplemental 5 D,E). We further examined autophagic transfer from the mitochondria to lysosomes using LysoTracker and MitoTracker. Our results showed that autophagic transmission was inhibited in the shOPA1 and shMFN2 groups compared with the vehicle group, manifested as less mitochondrial and lysosome colocalisation, which was not improved by EtBr (Fig. Supplemental 5 F,G). Therefore, we speculate that mitochondrial dysfunction during perfusion injury in diabetic myocardial ischaemia leads to mitoDNA spillover, and that mitoDNA deletion does not improve mitochondrial and lysosomal autophagy, indicating that mitoDNA release is downstream of autophagy and upstream of inflammatory signalling.

Next, to further clarify our hypothesis, it was shown that when cGAS-STING is activated, STING moves from the endoplasmic reticulum to the perinuclear Golgi and then conducts downstream signalling by phosphorylating TBK1 and IRF3. Immunofluorescence staining was performed to further demonstrate the activation of STING. The results showed that STING translocated from the cytoplasm to the perinuclear region (Fig. 6 G).

### STING inhibition abrogates inflammation response in diabetic MI/R injury

**Fig. 7:**
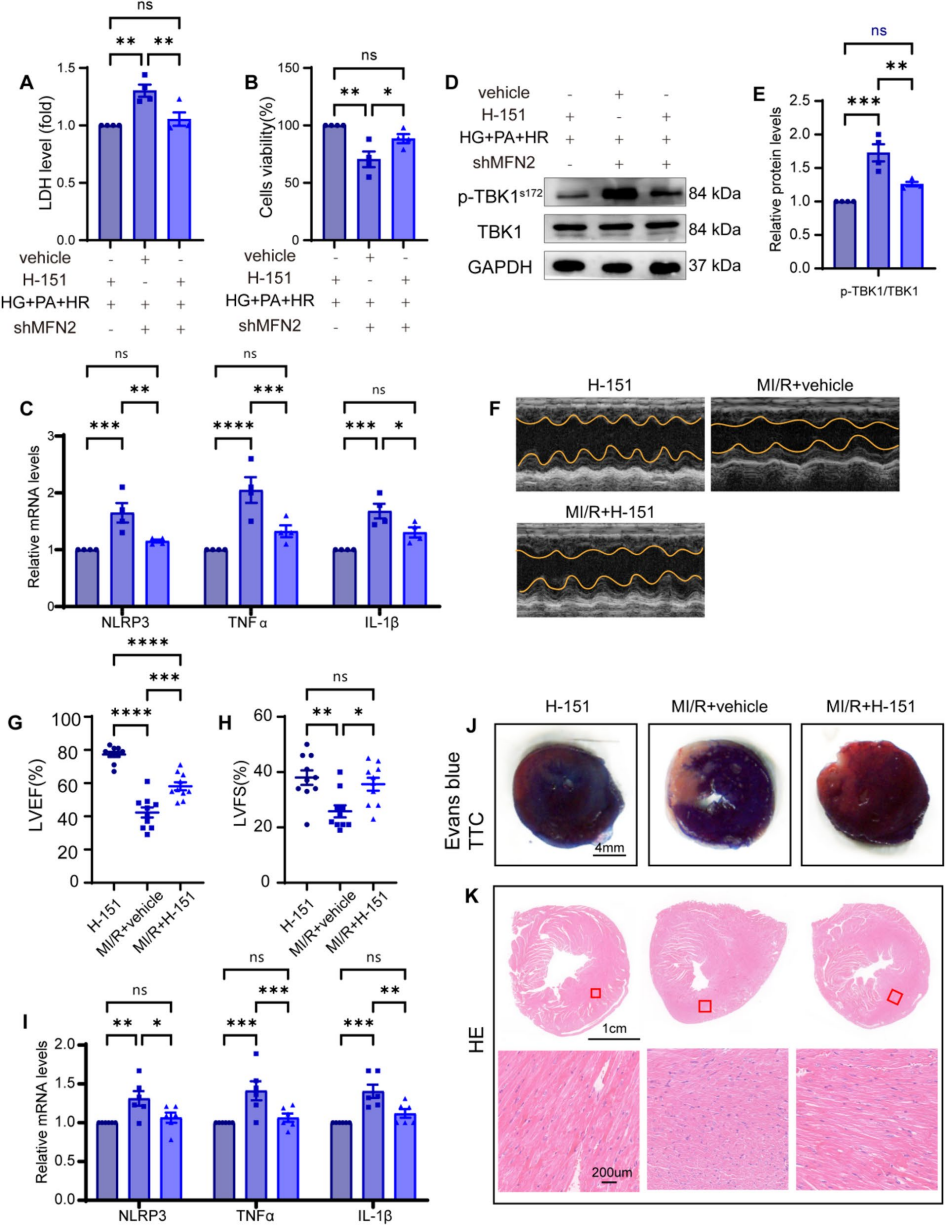
STING inhibition abrogates inflammation response in diabetic MI/R injury. **A** LDH level. **B** Cell viability. **C** and **I** mRNA levels of NLRP3, TNF-α, and IL-1β were detected using qRT-PCR. **D** Representative protein levels of p-TBK1^s172^ and TBK1 detected by western blotting. **E** Quantification of D. **F**–**H** Left ventricular ejection fraction and fractional shortening. (*n* = 10). **J** Representative images of Evans blue and TTC stained heart sections after MI/R or sham operation. Scale bar: 4 mm. **K** Representative images of HE-stained heart sections after MI/R or sham operation. Section scale bar: 1 μm. Magnified scale bar: 200 μm. All data are presented as the mean ± SEM. ^*^P < 0.05,^**^P < 0.01, ^***^P < 0.001,^****^P < 0.0001

Having found that mitoDNA leakage could trigger the cGAS-STING pathway, we investigated whether interfering with STING activity using H-151, a selective STING inhibitor which reduces TBK1 phosphorylation and inhibits STING palmitoylation, could exert protective effects against diabetic MI/R injury and provide potential clinical benefits. First, we assessed the effects of H-151 on diabetic MI/R injury and the inflammatory response in vitro. We observed that H-151 inhibition decreased LDH levels and increased cell viability compared to those in the vehicle-treated group (Fig. 7 A,B). In addition, H-151 treatment dramatically decreased the H9C2 cells’ ISFs mRNA levels (Fig. 7 C) and p-TBK1 protein expression compared to the vehicle-treated group (Fig. 7 D,E). The in vitro experiments suggested that H-151 inhibition repressed HG + PA + HR-induced cardiomyocyte injury and inflammation response.

**Fig. 8:**
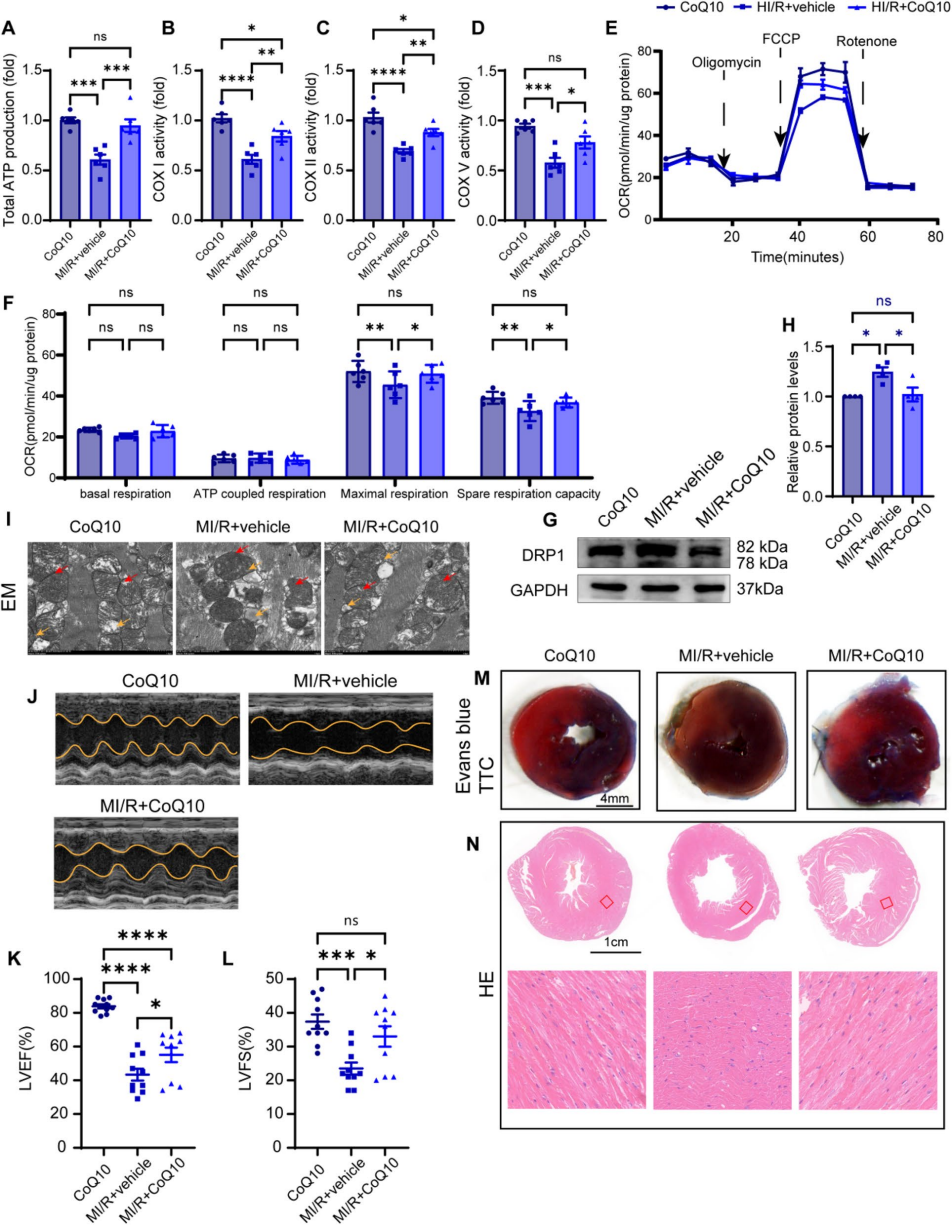
Therapeutic benefit of CoQ10 on mitochondrial function and restoring cardiac function in diabetic MI/R injury. **A**-**D** Total ATP production and ETC complex activity in cardiomyocytes. **E**–**F** Seahorse exhibited the alterations in respiratory capacity. **G** Representative protein level of DRP1 was detected by western blotting. **H** Quantification of H. All data are presented as mean ± SEM. ^*^P < 0.05, ^**^P < 0.01, ^***^P < 0.001, ^****^P < 0.0001. **I** Representative EM images of mitochondrial morphology. Scale bar: 10 μm. Yellow arrow: lipid droplets. Red arrow: damaged mitochondria. **J**-**K** Left ventricular ejection fraction and fractional shortening (*n* = 10). Data are presented as mean ± SEM. ^*^P < 0.05, ^***^P < 0.001, ^****^P < 0.0001. **L** Representative images of HE-stained heart sections after MI/R or sham operation. Section scale bar: 1 μm. Zoom scale bar: 200 μm. **M** Representative images of Evans blue and TTC stained heart sections after MI/R or sham operation. Scale bar: 4 mm

To further explore the therapeutic effects of STING inhibitors on diabetic MI/R injury in vivo, WT mice with sham or diabetic ischaemia–reperfusion injury were treated with H-151 or its vehicle control. Echocardiographic analysis revealed that H-151 supplementation increased the left ventricular ejection fraction and left ventricular fraction shortening compared with the MI/R-vehi group mice after ischaemia–reperfusion (Fig. 7 F–H). Furthermore, Evans-TTC staining showed that H-151 rescued myocardial infarction size in diabetic MI/R mice compared to vehicle mice (Fig. 8 J). The attenuation of the myocardial structure was also confirmed by HE staining (Fig. 8 K). This improved cardiac function in H-151-treated mice was consistent with the decreased ISFs mRNA expression compared with that in the vehicle group (Fig. 8 I). Taken together, these findings suggest that pharmacological H-151 inhibition can elicit functional and morphological benefits in diabetic MI/R injury, highlighting the potential therapeutic benefits for cardiac ischaemia reperfusion injury in diabetes.

### Therapeutic benefits of CoQ10 on mitochondrial function, reducing mitoDNA leakage and restoring cardiac function in diabetic MI/R injury

The aggravation of diabetic MI/R injury is related to mitochondrial dysfunction; therefore, we checked whether pharmacological CoQ10 can restore mitochondrial function and alleviate cardiac dysfunction. To determine whether CoQ10 could improve mitochondrial dysfunction in diabetic MI/R mice, we assessed the expression of ATP and mitochondrial ETC complexes and morphological changes in the mitochondria. Compared to the MI/R + vehi group, CoQ10 treatment significantly upregulated ATP production (Fig. 8 A) and ETC complex activity (Fig. 8 B-D). Seahorse analysis also revealed that CoQ10 rescued MI/R-induced mitochondrial dysfunction, with increased maximal respiration and spare respiratory capacity (Fig. 8 E,F). Compared to the MI/R + vehi group, CoQ10 treatment significantly downregulated DRP1 expression (Fig. 8 G,H). EM indicated that the MI/R+vehi group triggered more mitochondria with disorganised cristae structures, which could be improved by CoQ10 treatment (Fig. 8 I). Echocardiographic analysis revealed that CoQ10 administration did not adversely affect baseline cardiac function in WT mice. Moreover, CoQ10 treatment exerted protective effects in MI/R mice, as evidenced by a significant increase in the left ventricular ejection fraction and shortening of the left ventricular fraction compared with the MI/R-vehi group mice (Fig. 8 J-L). Histological analysis showed that CoQ10 rescued myocardial fibre integrity in diabetic MI/R mice compared to that in MI/R-vehi mice (Fig. 8 N). Furthermore, Evans-TTC staining showed that CoQ10 rescued myocardial infarction size in diabetic MI/R mice compared to that in MI/R-vehi mice (Fig. 8 M). Together, these data confirm that causes of increased MI/R vulnerability in diabetes involve impaired mitochondrial bioenergetics and that CoQ10 could rescue mitochondrial and cardiac dysfunction.

## Discussion

Diabetes is a global health problem. The mechanism of decreased tolerance to ischaemic heart disease is still unknown, and there is still no effective treatment. Therefore, it is important to elucidate the mechanism of MI/R deterioration in diabetes to improve cardiac function and prognosis. In the current study, we found that mitochondrial fusion was significantly reduced in HFD + STZ-induced diabetic MI/R injury, and mitochondrial fragmentation increased, leading to the accumulation of mitoDNA in the cytoplasm and activation of the cGAS-STING pathway, which triggered a series of inflammatory cascade responses that exacerbated MI/R injury. In contrast, we used the STING inhibitor H-151 to reduce the phosphorylation of TBK1 and inflammatory factors and improve cardiac function. In our interventional approach, we demonstrated that CoQ10, a fat-soluble antioxidant, attenuated cardiac function by restoring mitochondrial function and reducing mitochondrial fission. These results indicate that the restoration of mitochondrial function is critical for the heart. In conclusion, our study provides a new therapeutic approach for the treatment of diabetic MI/R injury.

In our study, we found that the levels of LDH, cTnT, CK-MB, and MDA were significantly increased in HDF + STZ-induced diabetic mice with MI/R injury, accompanied by a decrease in the mRNA levels of the antioxidant indicators SOD-1 and HO-1. Previous studies have shown that mitochondrial function is altered under these conditions [8, 29]. In our study, a decrease in mitochondrial fusion and an increase in fission were detected, accompanied by an accumulation of cytoplasmically damaged mitoDNA. This is consistent with previous studies showing that mitoDNA damage is exacerbated in the chronic ischaemic heart [30], and that changes in mitochondrial morphology may be key to cardiac remodelling [31]. Cytoplasmic mitoDNA-induced cGAS-STING activation plays a critical role in the pathogenesis of obesity-associated diabetic cardiomyopathy [32]. We found that a large amount of mitoDNA was present in the cytoplasm of cardiomyocytes with diabetic MI/R injury. To be more intuitive, we used H9C2 ventricular myocytes to construct a model of glycolipid toxicity, which was consistent with the in vivo results, and the cytoplasmic mitoDNA damage increased significantly. CoQ10 is a fat-soluble antioxidant that is widely available over-the-counter as a dietary supplement [33], although it is not currently *Food and Drug Administration* (FDA) approved for use in the treatment of any disease. Evidence suggests that CoQ10 attenuates ischaemic left ventricular systolic dysfunction by improving mitochondrial function [34, 35], lowering the risk of cardiovascular death [36]. Our evidence suggests that CoQ10 may attenuate the risk of HDF + STZ-induced diabetic MI/R injury by improving mitochondrial fusion.

Recent studies have identified pattern recognition receptors (PRRs), including TLR-9, the NLRP3 inflammasome, and the cGAS-STING pathway, as key promoters of mitoDNA-associated inflammatory responses [37]. As expected, we found that the expression of the key DNA sensor protein in the cGAS-STING pathway was upregulated in diabetic MI/R injury mice, phosphorylating and activating its downstream effector proteins TBK1 and IRF3. It also released a large number of pro-inflammatory factors, including NLRP3, TNFα, and IL-1β. Therefore, we speculated that diabetic MI/R reduced mitochondrial fusion, increased mitochondrial fission, induced mitoDNA to escape into the cytoplasm, and activated the cGAS-STING signalling pathway to cause inflammation. To verify the role of mitochondrial fusion in cardiac function, we constructed mice with cardiac OPA1^+/+^ and MFN2^+/+^ overexpression. Surprisingly, our results demonstrated that MFN2^+/+^, but not OPA1^+/+^, reduced mitoDNA damage by improving mitochondrial fusion. Mitochondrial fusion is regulated by different proteins, including mitochondrial outer membrane MFN1 and MFN2 and mitochondrial inner membrane OPA1. MFN1 and MFN2 are specifically expressed in many tissues, with MFN1 being ubiquitously expressed in the heart and testis, whereas MFN2 levels are increased in the heart, skeletal muscle, and brain, which could explain the differential roles of these proteins. Previous studies have reported reduced dendritic growth, spine formation, and cell survival in Purkinje cells of mice conditional on Mfn2^−/−^ in the cerebellum, whereas Mfn1^−/−^ mice showed no such defects [38]. Another study also reported that T2D is associated with reduced expression of MFN2, possibly by affecting mitochondrial function in skeletal muscles [39]. Our study found significantly improved mitochondrial biogenesis in MI/R in MFN2^+/+^ mice compared with control mice, as evidenced by increased mitochondrial ATP generation and mitochondrial ETCs activity.

It has been suggested that mitochondrial fusion may protect damaged mitoDNA through content mixing, allowing genomes with different mitoDNAs to complement each other [40], and mitochondrial fusion is required for skeletal muscle mitochondrial stability and mitoDNA mutation tolerance [41]. This would support our finding that mitochondrial fusion was reduced and damaged mitoDNA was increased and enriched in the cytoplasm during diabetic MI/R injury. cGAS-STING signalling plays an important regulatory role in microbial and tumour immunology through the induction of cytokines, mainly type I interferons. Recently, abnormal and disordered signalling of the cGAS-STING axis has been closely associated with various sterile inflammatory diseases, including heart failure, myocardial infarction, and cardiac hypertrophy [13, 42]. This is because a large number of DAMPs (mitoDNA and DNA in extracellular vesicles) are released from repeated damage to metabolic organelles and tissues, which can be sensed by the cGAS-STING pathway, inducing a strong type I interference priming reaction. However, the effect of excess type I interferon signalling on cardiovascular and metabolic health remains unclear. Our in vivo study found that mitoDNA was enriched in the cytoplasm and that the cGAS-STING pathway was activated in the hearts of diabetic MI/R-injured mice. In terms of the mechanism, we used H9C2 cardiomyocytes to construct shOPA1 and shMFN2, and found that after H/R was induced by glycolipids, the damaged mitoDNA increased significantly, and the cGAS-STING pathway was strongly activated. After EtBr treatment, mitoDNA was significantly reduced, the cGAS-STING pathway was inhibited, and myocardial inflammation and injury were significantly reduced. It is worth noting that in our in vitro study, we detected mitophagy and lysophagy and found that EtBr treatment did not improve mitochondrial or lysosomal autophagy after H/R injury induced by glycolipid toxicity. Whether this means that the release of mitoDNA is downstream of autophagy requires further exploration. Recent studies have reported that disruption of mitophagy during acute liver injury promotes the extracellular release of mitoDNA, activates the NLRP3/caspase-1/GSDMD pathway, and exacerbates pyroptosis in hepatocytes [43]. In contrast, previous studies have reported that changes in STING localisation are important for specific activation steps in the TBK1-IRF3 signalling pathway [44]. Our in vitro study found that after glycolipid-induced H/R injury, STING accumulates around the nucleus in large quantities, and this phenomenon can be inhibited by EtBr. In vivo studies have shown that the STING inhibitor H-151 can reduce diabetic MI/R injury, which provides a new approach for the treatment of diabetic MI/R injury.

Although our study proposes a new hypothesis, it provides a new target for the treatment of diabetic MI/R. However, there are some limitations to our study. First, mitochondrial dynamics include mitochondrial fusion, fission, mitophagy, and mitochondrial biogenesis. Although we have proven that mitoDNA is caused by the reduction of mitochondrial fusion, the complex mechanism requires further study. Second, mitochondrial fusion comprises OPA1, which mediates inner membrane fusion, and MFN1 and MFN2, which mediate outer membrane fusion. We found that the role of MFN2 may be greater than that of OPA1; however, the redundant role of mitochondrial fusion proteins needs to be further clarified. Third, diabetes itself contributes to the complexity of the disease.

Taken together, this study revealed that decreased mitochondrial fusion-mediated mitoDNA cytoplasmic enrichment, activation of the cGAS-STING pathway, and phosphorylation of the TBK1-IRF3 pathway are important contributors to MI/R injury in diabetes. Given the profound clinical importance of diabetic MI/R and mitochondrial function, our findings demonstrate the importance of the mitoDNA-induced cGAS-STING pathway as a potential therapeutic target for the treatment of MI/R injury in diabetic heart disease.

### Availability of data and materials

The datasets supporting the conclusions of this article are included within the article (and its Additional files).

### Supplementary Information

**Additional file 1. **

**Additional file 2: Supplemental 1.** The content of mitoDNA in serum. (A) The mRNA levels of Dloop1, Dloop2, Dloop3, CytB, Rnr2, ND2, and ND4 in serum were detected using qRT-PCR. Data are presented as the mean ± SEM. ^*^P < 0.05 versus ND+sham group; ^#^*P* < 0.05 versus ND+MI/R group; ^&^*P* < 0.05 versus HFD+STZ +sham group. **Supplemental 2.** (A) Representative immunoblot images showing OPA1 and MFN2 protein expression levels. (B) Quantification of A. (C) Representative immunoblotting images showing PINK1, Parkin, LC3 II, and P62 protein expression. (D) Quantification of C. Data are presented as the mean ± SEM. ^**^P < 0.01, ^***^P < 0.001, ^****^P < 0.0001. **Supplemental 3.** PA drives cardiomyocyte mitochondrial dysfunction in vitro. Each group was exposed to hypoxia for 4 h and reoxygenation for 2 h. (A) Schematic diagram of H9C2 cell experiment, NS (low glucose: 5 mM), HG (high glucose: 25 mM), and PA (palmitate: concentrations of 0, 200, 400, 800, and 1600 µM). (B) ATP content of the supernatant. (C) ATP content of cells. (D-F) ETC complex activity. Data are presented as the mean ± SEM. (G-H) Protein levels of Cyto C were detected using western blotting. Data are presented as the mean ± SEM. **Supplemental 4.** HG+PA+HR causes mitoDNA to escape into the cytoplasm. (A)The mRNA levels of Dloop1, Dloop2, Dloop3, CytB, Rnr2, ND2, and ND4 in H9C2 cells were detected using qRT-PCR. Data are presented as the mean ± SEM. ^***^P < 0.001. **Supplemental 5.** (A) Representative immunoblot images showing MFN2 and OPA1 protein expression levels. (B) Quantification of A. Data are presented as the mean ± SEM. ^***^P < 0.001. (C) Representative EM images of mitochondrial morphology in each group. (D) Representative fluorescence images of mitophagy in each group. (E) Quantification of D. (F) Representative fluorescent images of lysophagy in each group. (G) Quantification of F.

## Data Availability

The datasets generated during and analysed during the current study are available from the corresponding author on reasonable request.

## Abbreviations

AAR: Area at risk
AUC: Area under the curve
DAMP: Damage-associated molecular patterns
EM: Electron microscope
EtBr: Ethidium bromide
ETC: Electron transport chain
HFD: High-fat diet
HG: High glucose
IPGTT: Intraperitoneal glucose tolerance test
ISF: Inflammation-stimulated factors
ITT: Insulin tolerance test
LVEF: Left ventricular ejection fraction
LVFS: Left ventricular shortening fraction
LVID: Left ventricular internal dimension
MI/R: Myocardial ischaemia–reperfusion
mitoDNA: Mitochondrial DNA
ND: Normal caloric diet
NIH: National Institutes of Health
NMVM: Neonatal mouse ventricular myocytes
NRVM: Neonatal mouse ventricular myocyte
OCR: Oxygen consumption rate
PA: Palmitic acid or palmitate
PRR: Pattern recognition receptors
ROS: Reactive oxygen species
STING: Stimulation of the interferon gene
STZ: Streptozocin
