# Supplementary material for: Decreased MFN2 activates the cGAS-STING pathway in diabetic myocardial ischaemia–reperfusion by triggering the release of mitochondrial DNA
Source: Cell Commun Signal. 2023 Aug 3;21:192. doi: 10.1186/s12964-023-01216-y (PMC10398939; doi:10.1186/s12964-023-01216-y)
Supplement: Supplementary file 3 — Additional file 2: Supplemental 1. The content of mitoDNA in serum. (A) The mRNA levels of Dloop1, Dloop2, Dloop3, CytB, Rnr2, ND2, and ND4 in serum were detected using qRT-PCR. Data are presented as the mean ± SEM. *P < 0.05 versus ND+sham group; #P < 0.05 versus ND+MI/R group; &P < 0.05 versus HFD+STZ +sham group. Supplemental 2. (A) Representative immunoblot images showing OPA1 and MFN2 protein expression levels. (B) Quantification of A. (C) Representative immunoblotting images showing PINK1, Parkin, LC3 II, and P62 protein expression. (D) Quantification of C. Data are presented as the mean ± SEM. **P < 0.01, ***P < 0.001, ****P < 0.0001. Supplemental 3. PA drives cardiomyocyte mitochondrial dysfunction in vitro. Each group was exposed to hypoxia for 4 h and reoxygenation for 2 h. (A) Schematic diagram of H9C2 cell experiment, NS (low glucose: 5 mM), HG (high glucose: 25 mM), and PA (palmitate: concentrations of 0, 200, 400, 800, and 1600 µM). (B) ATP content of the supernatant. (C) ATP content of cells. (D-F) ETC complex activity. Data are presented as the mean ± SEM. (G-H) Protein levels of Cyto C were detected using western blotting. Data are presented as the mean ± SEM. Supplemental 4. HG+PA+HR causes mitoDNA to escape into the cytoplasm. (A)The mRNA levels of Dloop1, Dloop2, Dloop3, CytB, Rnr2, ND2, and ND4 in H9C2 cells were detected using qRT-PCR. Data are presented as the mean ± SEM. ***P < 0.001. Supplemental 5. (A) Representative immunoblot images showing MFN2 and OPA1 protein expression levels. (B) Quantification of A. Data are presented as the mean ± SEM. ***P < 0.001. (C) Representative EM images of mitochondrial morphology in each group. (D) Representative fluorescence images of mitophagy in each group. (E) Quantification of D. (F) Representative fluorescent images of lysophagy in each group. (G) Quantification of F. [file 12964_2023_1216_MOESM2_ESM.docx]

**
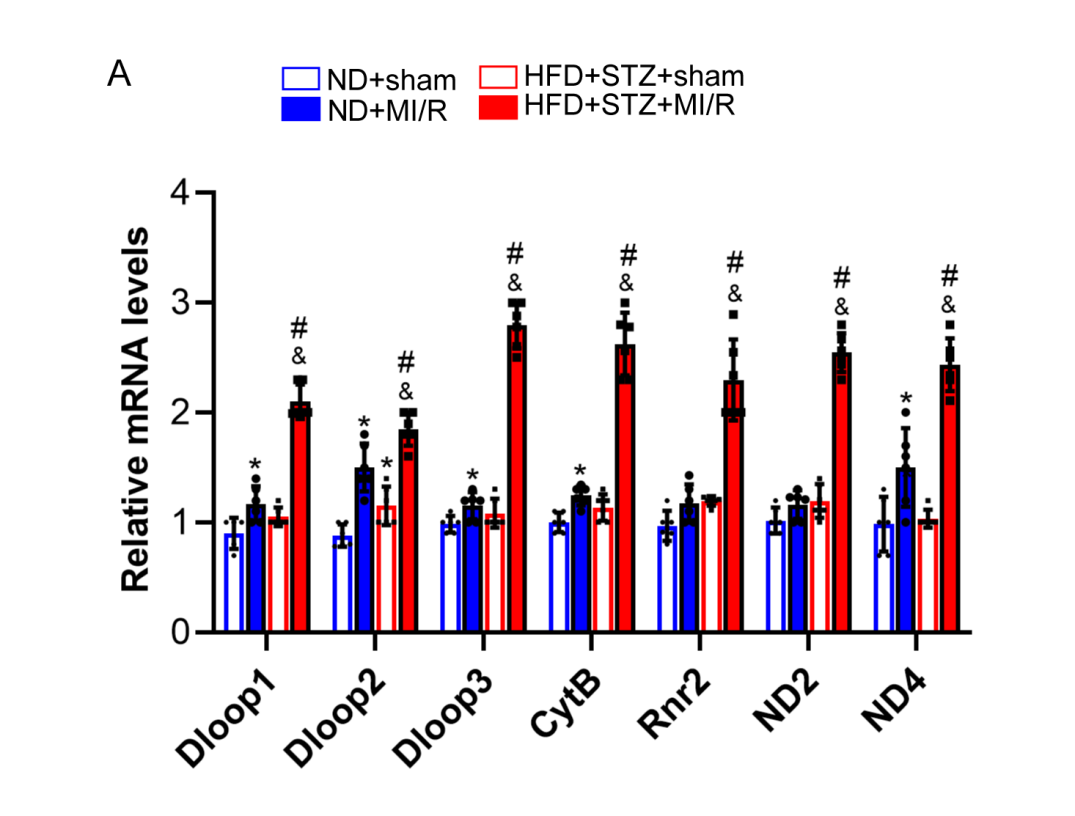
**

**Supplemental 1. The content of mitoDNA in serum**

1. The mRNA levels of Dloop1, Dloop2, Dloop3, CytB, Rnr2, ND2, and ND4 in serum were detected using qRT-PCR. Data are presented as the mean ± SEM. ^*^*P* < 0.05 versus ND+sham group; ^#^*P* < 0.05 versus ND+MI/R group; ^&^*P* < 0.05 versus HFD+STZ +sham group.


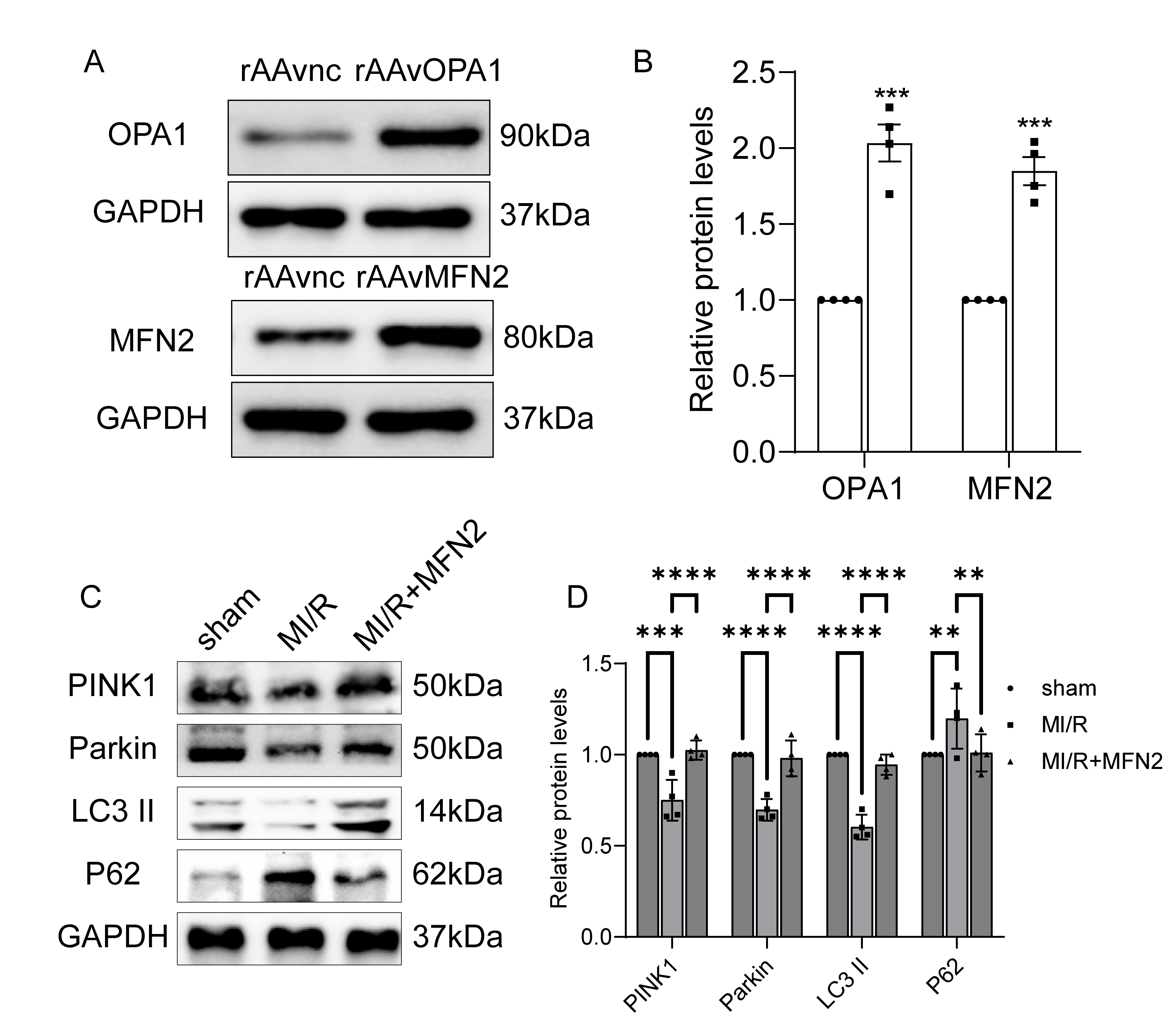


**Supplementa**l **2**. (A) Representative immunoblot images showing OPA1 and MFN2 protein expression levels. (B) Quantification of A. (C) Representative immunoblotting images showing PINK1, Parkin, LC3 II, and P62 protein expression. (D) Quantification of C. Data are presented as the mean ± SEM. ^**^*P* < 0.01, ^***^*P* < 0.001, ^****^*P* < 0.0001.


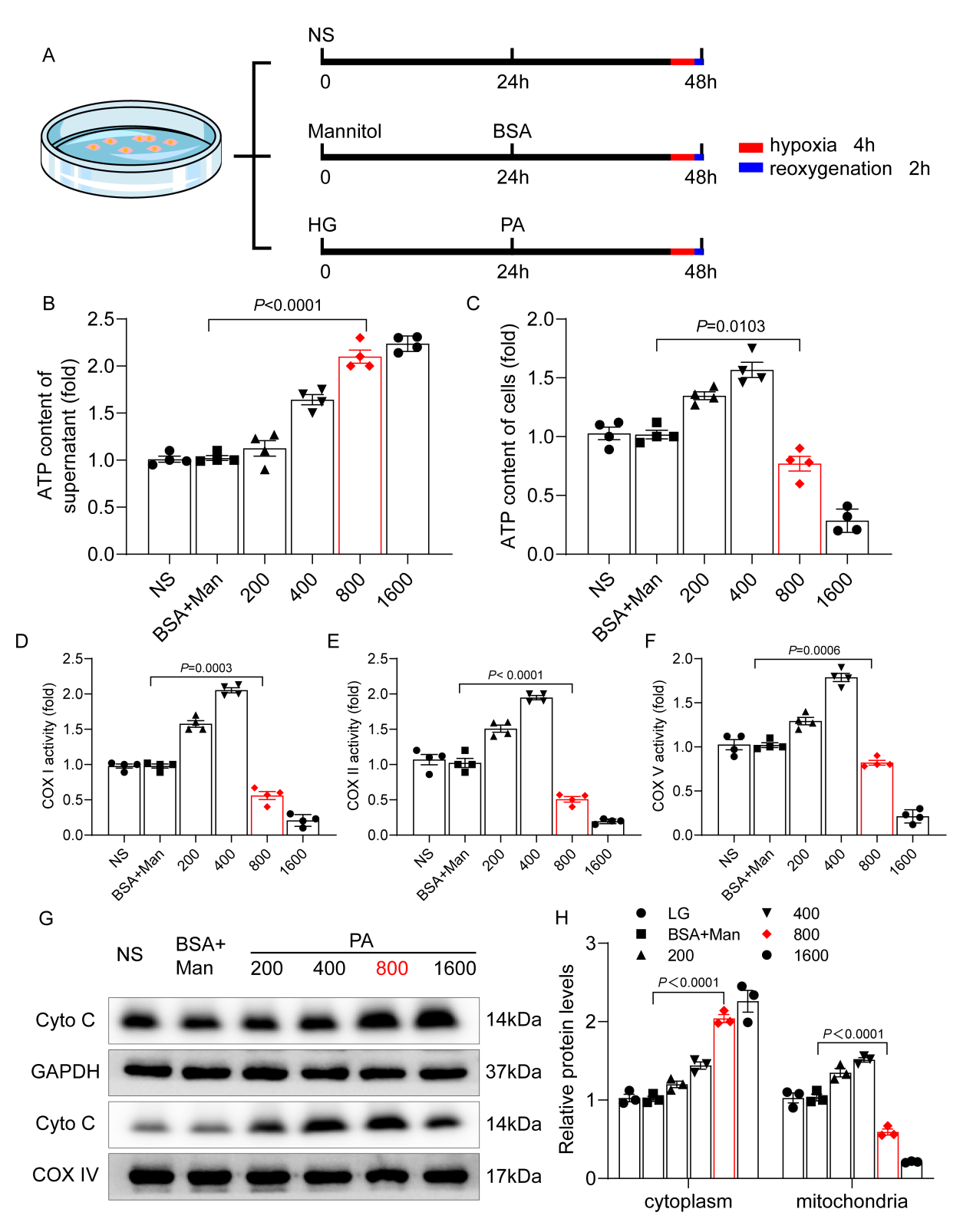


**Supplemental 3. PA drives cardiomyocyte mitochondrial dysfunction in vitro**

Each group was exposed to hypoxia for 4 h and reoxygenation for 2 h. (A) Schematic diagram of H9C2 cell experiment, NS (low glucose: 5 mM), HG (high glucose: 25 mM), and PA (palmitate: concentrations of 0, 200, 400, 800, and 1600 µM). (B) ATP content of the supernatant. (C) ATP content of cells. (D-F) ETC complex activity. Data are presented as the mean ± SEM. (G-H) Protein levels of Cyto C were detected using western blotting. Data are presented as the mean ± SEM.

**
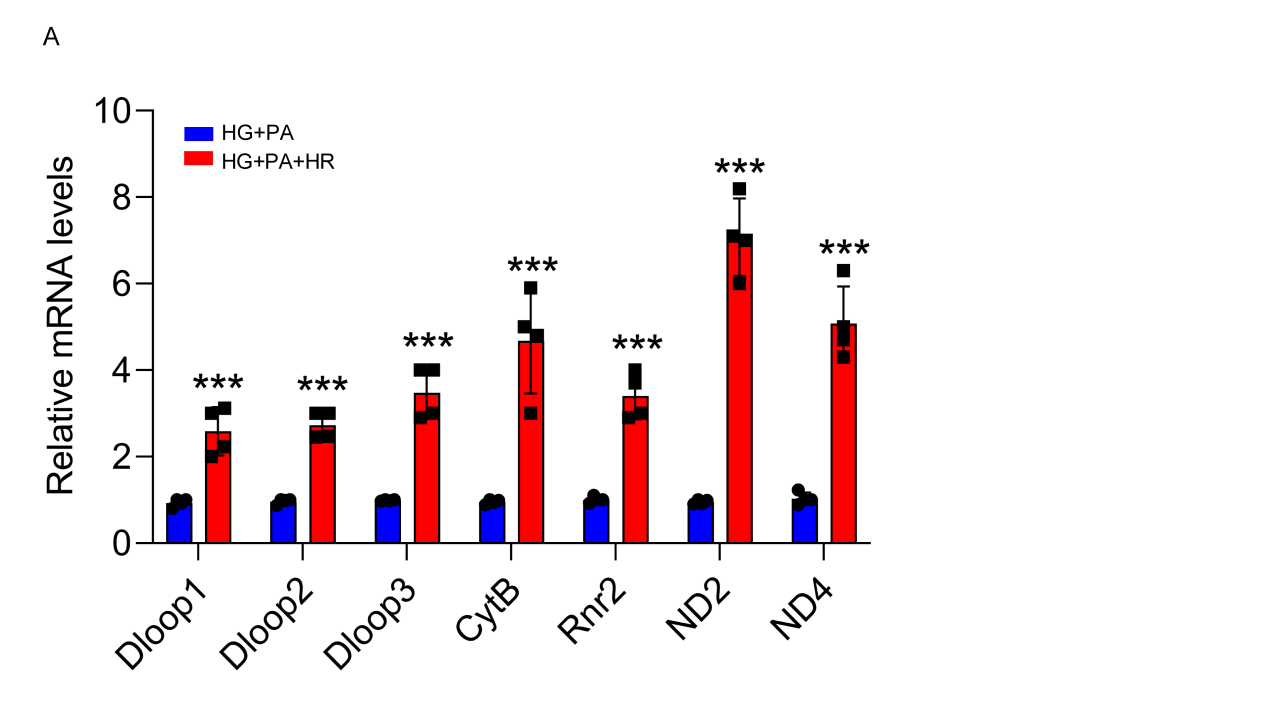
**

**Supplemental 4. HG+PA+HR causes mitoDNA to escape into the cytoplasm**

(A)The mRNA levels of Dloop1, Dloop2, Dloop3, CytB, Rnr2, ND2, and ND4 in H9C2 cells were detected using qRT-PCR. Data are presented as the mean ± SEM. ^***^*P* < 0.001.

**
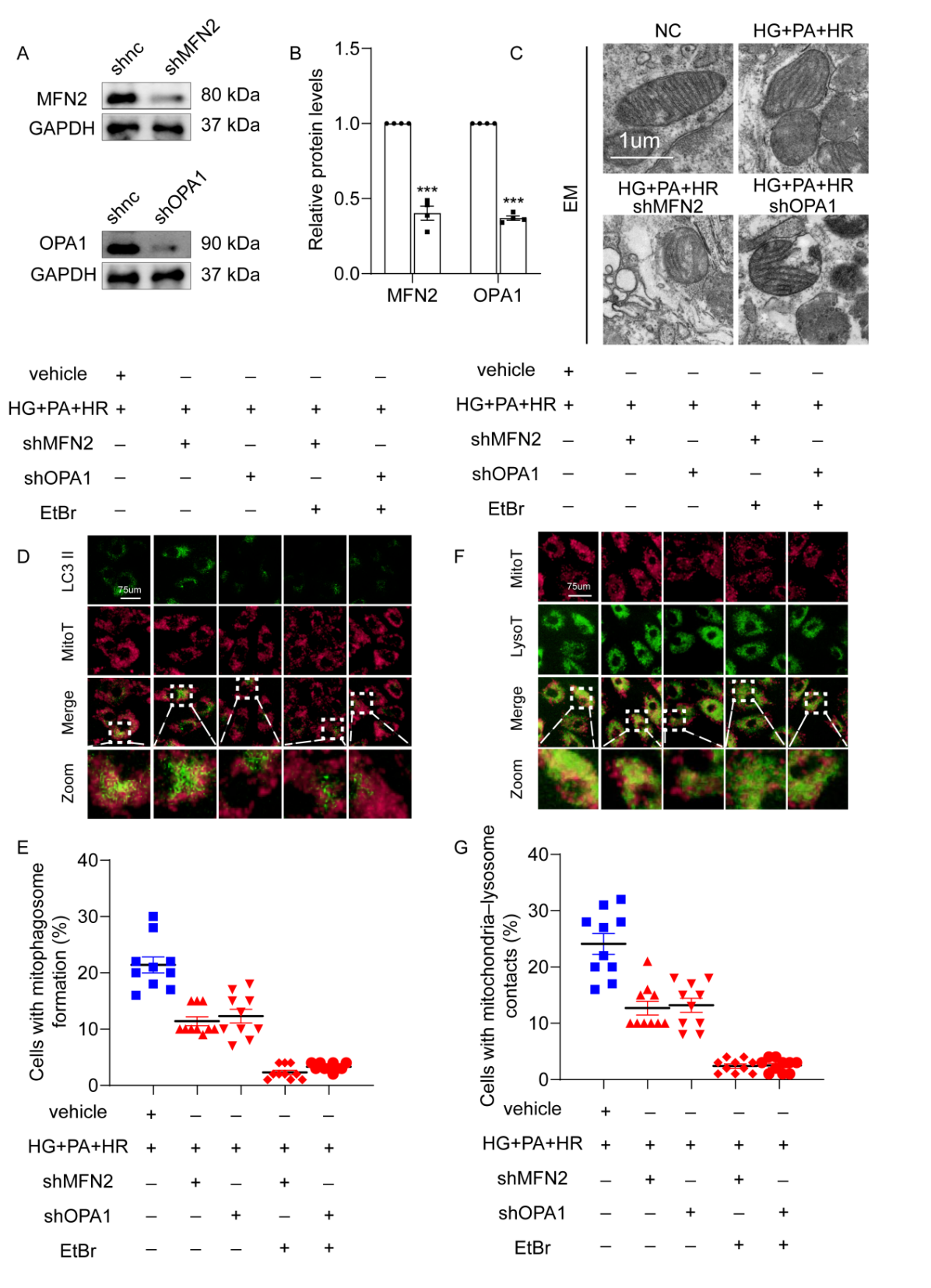
**

**Supplemental 5.** (A) Representative immunoblot images showing MFN2 and OPA1 protein expression levels. (B) Quantification of A. Data are presented as the mean ± SEM. ^***^*P* < 0.001. (C) Representative EM images of mitochondrial morphology in each group. (D) Representative fluorescence images of mitophagy in each group. (E) Quantification of D. (F) Representative fluorescent images of lysophagy in each group. (G) Quantification of F.
